# Functionalized MoS_2_-nanosheets with NIR-Triggered nitric oxide delivery and photothermal activities for synergistic antibacterial and regeneration-promoting therapy

**DOI:** 10.1186/s12951-023-02167-9

**Published:** 2023-12-04

**Authors:** Zhixiang Mu, Ting Jin, Tengda Chu, Hongyang Lu, Yuanqi Chen, Sisi Li, BaiRui Zeng, Chen Huang, Kezheng Lei, Xiaojun Cai, Hui Deng, Rongdang Hu

**Affiliations:** School and Hospital of Stomatology, Wenzhou Medical University, Wenzhou, Zhejiang 325027 P.R. China

**Keywords:** NO gas therapy, Photothermal therapy, Antibacterial, Angiogenesis, Promoting wound healing

## Abstract

Bacterial infection in skin and soft tissue has emerged as a critical concern. Overreliance on antibiotic therapy has led to numerous challenges, including the emergence of multidrug-resistant bacteria and adverse drug reactions. It is imperative to develop non-antibiotic treatment strategies that not only exhibit potent antibacterial properties but also promote rapid wound healing and demonstrate biocompatibility. Herein, a novel multimodal synergistic antibacterial system (SNO-CS@MoS_2_) was developed. This system employs easily surface-modified thin-layer MoS_2_ as photothermal agents and loaded with S-nitrosothiol-modified chitosan (SNO-CS) via electrostatic interactions, thus realizing the combination of NO gas therapy and photothermal therapy (PTT). Furthermore, this surface modification renders SNO-CS@MoS_2_ highly stable and capable of binding with bacteria. Through PTT’s thermal energy, SNO-CS@MoS_2_ rapidly generates massive NO, collaborating with PTT to achieve antibacterial effects. This synergistic therapy can swiftly disrupt the bacterial membrane, causing protein leakage and ATP synthesis function damage, ultimately eliminating bacteria. Notably, after effectively eliminating all bacteria, the residual SNO-CS@MoS_2_ can create trace NO to promote fibroblast migration, proliferation, and vascular regeneration, thereby accelerating wound healing. This study concluded that SNO-CS@MoS_2_, a novel multifunctional nanomaterial with outstanding antibacterial characteristics and potential to promote wound healing, has promising applications in infected soft tissue wound treatment.

**Supplementary Information:**

The online version contains supplementary material available at 10.1186/s12951-023-02167-9.

## Introduction

In clinical dermatology, skin and soft tissue infections (SSTIs) are prevalent infectious disorders [1]. Open skin wounds are susceptible to bacterial invasion, resulting in wound infections that can lead to multiple adverse effects [2]. In mild cases, it delays tissue healing, leading to disease chronicity and an increased medical burden. In more extreme instances, life-threatening systemic problems may occur [3, 4]. Antibiotic therapy is currently the primary treatment strategy for SSTIs. Nevertheless, antibiotics used for SSTIs have certain limitations. First, the anti-infective effects of antibiotic therapy are limited. Microorganisms are prone to acquiring multidrug resistance due to antibiotic misuse and abuse [5, 6], which has emerged as a major public health threat in the 21st century [7]. Antibiotic overuse can cause toxic and side effects on the body [8], such as liver injury [9, 10] and liver failure [11, 12]. In addition to failing to promote early wound closure, using antibiotics alone in SSTIs treatment prolongs the period of wound care and increases the risk of secondary infection. Therefore, there is an urgent need to develop highly antibacterial, non-antibiotic treatment strategies that facilitate the rapid recovery of infected wounds and possess good biocompatibility.

Photothermal therapy (PTT) is a novel approach that utilizes photothermal agents (PTAs) to convert the optical energy of near-infrared (NIR) light into localized physical heat energy. This heat energy is effective in antibacterial therapy as it causes bacterial cell membrane rupture and protein denaturation. PTT has gradually emerged as an ideal therapeutic strategy to replace antibiotic therapy [13] due to its broad-spectrum antibacterial activity, absence of multidrug resistance [14], and deeper tissue penetration with good biosafety of NIR light [15]. Among the representative 2D transition metal sulfides, Molybdenum (IV) disulfide (MoS_2_) has gained recognition as one of the best candidates for photothermal antibacterial agents owing to its good biocompatibility, high photothermal conversion efficiency, large surface area, and easy surface modification [16]. However, using MoS_2_ alone for PTT also leads to several key problems. (1) Poor photothermal stability of MoS_2_ nanosheets under physiological conditions significantly restricts their application in vivo. (2) Low concentration of MoS_2_ exhibit limited heating performance. To achieve optimal antibacterial effects, a high concentration of MoS_2_ is often required to sustain local temperatures of 70 °C and above for an extended duration of thermotherapy [17]. However, it’s worth noting that most histocytes have lower heat resistance compared to bacteria [18, 19]. Consequently, PTT relying on a high concentration of MoS_2_ carries a substantial risk of damaging surrounding healthy tissues. (3) MoS_2_ photothermal therapy can disrupt the microvasculature, thereby affecting the healing process of infected wounds. To address these issues, numerous studies have explored various approaches such as hydrophobic modification [20], lysozyme functionalization [21] and antibiotic loading [22] on the surface of MoS_2_ nanosheets to achieve multimodal synergistic low-temperature PTT. However, these approaches continue to face obstacles, including their inability to promote rapid wound healing and low biocompatibility. Therefore, there is an urgent need to investigate a novel synergistic therapeutic system that can enhance the antibacterial impact of MoS_2_ PTT while significantly improving the healing of infected wounds with good biocompatibility.

Gas therapy is a novel strategy that uses endogenous signaling molecules for disease treatment [23]. It has been extensively researched in the biomedical field due to its multiple advantages, including high efficiency, safety, low toxicity, and minimal side effects [24]. Nitric oxide (NO) is considered a highly effective broad-spectrum antibacterial agent that does not exhibit drug resistance. It can directly kill bacteria through a series of unique pathways. Unlike the antibacterial mechanism of conventional antibiotics [25], high concentrations of NO react with superoxide (O_2_^-^) to generate peroxynitrite (ONOO^-^), leading to rapid bacterial apoptosis through DNA deamidation, lipid peroxidation [26, 27] and abnormal protein function caused by S-nitrosylation [28, 29]. The current study indicated that NO molecules can synergize with PTT to achieve effective antibacterial activity [30]. In addition to its antibacterial effects, low concentrations of NO have also been shown to promote rapid wound healing. The mechanism can be summarized as follows: (1) triggering and transducing of cell proliferation and differentiation through the activation of mitogen-activated protein kinase and fibroblast proliferation [31]; (2) mediating angiogenesis by promoting endothelial cell proliferation and expressing pro-angiogenic factors [32]; (3) enhancing peripheral tissue nutrient supply by improving blood flow, increasing vascular permeability [33] and inducing wound epithelization by promoting fibroblasts migration and proliferation at the wound site, thereby significantly expediting the repair and healing of diseased tissues. In conclusion, based on the excellent performance of NO gas therapy in antibacterial and promoting the rapid healing of infected wounds, We predicted that the combination of NO gas therapy and MoS_2_ photothermal antibacterial therapy could establish a non-antibiotic therapeutic system that not only has an antibacterial effect but also accelerates the rapid healing of infected wounds.

Although NO gas therapy can enhance the antibacterial activity of PTT and significantly accelerate the healing process of infected wounds, its gas properties, short half-life (~ 5 s), and limited diffusion radius (~ 200 μm) [34] pose challenges for the controlled release of NO at the site of lesion. To address the issue of controlled NO delivery, several NO gas donor molecules have been developed. These donors can release NO in response to specific stimuli, such as high temperature [35], ascorbic acid [36] and enzymes [37]. However, achieving controlled NO release remains difficult due to the widespread availability of ascorbic acid and enzymes in vivo. In comparison to ascorbic acid- and enzyme-mediated NO release, photothermal-mediated NO release has attracted extensive attention because of its superior temporal and spatial control [25]. S-nitrosothiol (RSNO) is a new small-molecule NO gas donor that can rapidly generate NO via photothermal responsiveness [38]. Furthermore, under physiological conditions in vivo, RSNO can spontaneously break down slowly and yield low concentrations of NO molecules. These characteristics indicate that RSNO has the potential to synergistically enhance antibacterial activity, promote angiogenesis, and expedite wound healing through the controlled release of NO.

**Scheme 1:**
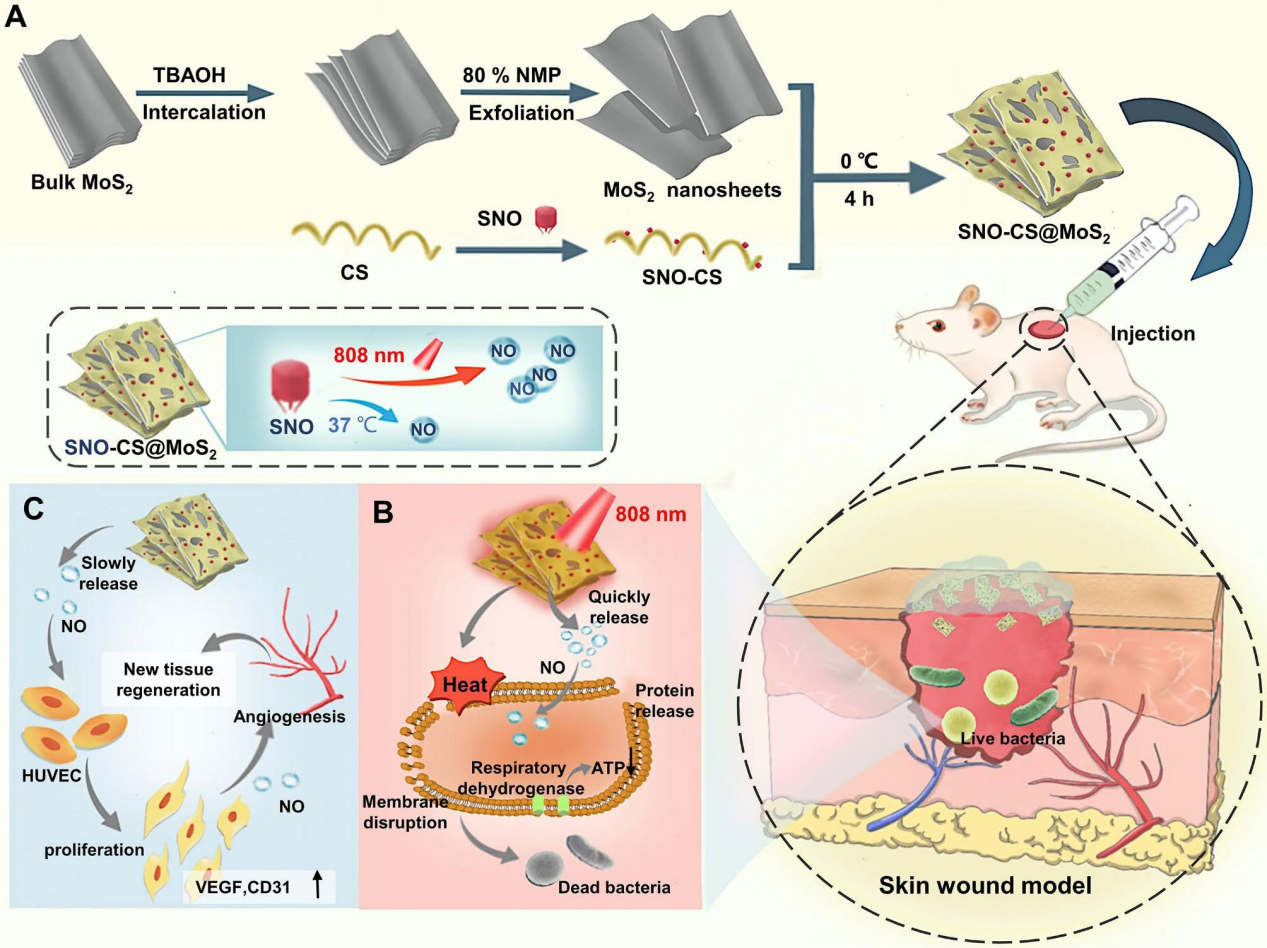
**A**) the preparation of SNO-CS@MoS_2_; **B**) PTT- controlled rapid release of a large amount of NO synergized with PTT for efficient antibacterial activity; **C**) stable slowly release of trace amount of NO under physiological conditions promotes fibroblasts migration, proliferation and vascularization

In conclusion, a novel multimodal synergistic antibacterial system can be developed by combining RSNO with MoS_2_, which harnesses the therapeutic potential of NO gas therapy and MoS_2_ PTT. To efficiently combine RSNO with MoS_2_ and further improve MoS_2_ stability under physiological conditions, this study first chemically grafted RSNO onto chitosan (CS) to prepare SNO-modified chitosan (SNO-CS). Subsequently, SNO-CS was electrostatically adsorbed onto the surface of mono- and few-layer MoS_2_ nanosheets to successfully construct SNO-CS@MoS_2_ (Scheme 1 A). Owing to the ingenious combination of the excellent performances of MoS_2_, PTT, and NO gas therapy, we hypothesized that SNO-CS@MoS_2_ will exhibit the following characteristics: (1) superior photothermal stability and bacterial binding properties; (2) PTT-controlled rapid release of a large amount of NO, and stable slow release of trace amounts of NO under physiological conditions; (3) high concentration of NO synergized with PTT for efficient antibacterial activity; (4) low concentration of NO significantly promotes fibroblasts migration, proliferation, and vascularization (Scheme 1B**-C**).

## Materials and methods

### Materials

MoS_2_ was acquired from Nanjing XFNANO Materials Tech Co. Ltd. CS (Deacetylation degree 90%) was obtained from Shanghai yuanye Bio-Technology Co. Ltd. Tetrabutylammonium hydroxide (~ 25% in H_2_O), N-(3-Dimethylaminopropyl)-N′-ethyl carbodiimide hydrochloride (EDCI), *p*-toluenesulfonic acid, 3-mercaptopropionic acid, methanol, N-hydroxy succinimide (NHS), N-methylpyrrolidone (NMP), N, N-dimethylformamide (DMF), and tert-butyl nitrite were purchased from Aladdin Biochemical Technology Co., Ltd. (China).

Mouse fibroblasts (L929), Human umbilical vein endothelial cells (HUVECs), *Escherichia coli* (*E. coli*, ATCC 25,923), and *Staphylococcus aureus* (*S. aureus*, ATCC 25,922) were obtained from the American Type Culture Collection (ATCC). Methyl thiazolyl diphenyl-tetrazolium bromide (MTT), Griess reagent kit, ATP assay kit, BCA Protein assay kit, 3-Amino,4-aminomethyl-2’,7’-difluorescein diacetate (DAF-FM DA), and Calcein/PI cell viability/cytotoxicity assay kit were purchased from beyotime biotechnology (China). Live/dead bacteria double staining kit was obtained from Shanghai Beibo Biotechnology Co. Ltd. Mouse anti-CD31 and anti-VEGFα were bought from proteintech (China).

### Preparation and characterization of MoS_2_ nanosheets

MoS_2_ nanosheets were prepared by liquid-phase exfoliation. First, bulk MoS_2_ was dispersed in a tetrabutylammonium hydroxide (TBAOH) solution at a concentration of 5 mg mL^-1^ and stirred overnight at room temperature to reduce the interlayer forces between the MoS_2_ sheets. Afterwards, the bulk MoS_2_ was rinsed twice with ethanol to remove residual TBAOH. The resulting precipitate was collected in a 50 mL centrifuge tube and dispersed in an 80% aqueous solution of NMP. The precipitate was then completely dispersed through bath sonication, and subsequently exfoliated into MoS_2_ nanosheets using probe sonication (JY92-IIDN, Scientz) for 10 h with a 3 s on/off cycle. The solution was centrifuged for 45 min at 5000 rpm to remove any unexfoliated MoS_2_ particles. The resultant supernatant, containing the exfoliated nanosheets, was further centrifuged at 15,000 rpm for 30 min to remove excess organic solvent. Finally, the obtained precipitate was re-dispersed in deionized water, resulting in a transparent and homogeneous dark-green solution of MoS_2_ nanosheets. The structure of MoS_2_ nanosheets was characterized using a UV-visible photometer (Ultrospec 7000, Biochrom) and Raman spectrometer (Horiba Evolution, HORIBA Scientific). The surface morphology of MoS_2_ nanosheets was observed using scanning electron microscopy (SEM, SU8010, HITACHI) and transmission electron microscopy (TEM, FEI Tecnai G2 F20, Thermo Fisher). The particle size and zeta potential of MoS_2_ were analyzed using a malvern particle size analyzer (Zetasizer Nano ZS-90, Malvern Panalytical).

### Preparation and characterization of SNO-CS@MoS_2_

As previously reported, 3-(nitroso) propionic acid (SNO) was initially produced prior to the synthesis of SNO-CS [39]. Briefly, an excess of tert-butyl nitrite (4 mmol) and 212 mg 3-mercaptopropionic acid were mixed in a 2 mL solution of DMF, and the reaction mixture was kept in an ice bath under a nitrogen atmosphere for 6 h. Afterward, any unreacted tert-butyl nitrite was removed by vacuuming for 1 h to obtain the SNO solution. The distinctive peak of SNO was characterized using a UV-visible photometer.

To prepare organic solvent-soluble CS, *p*-toluene sulfonic acid and CS were dissolved in 5 mL of deionized water at a CS concentration of 10 mg mL^-1^. The resulting solution was stirred overnight at room temperature. The undissolved CS was then removed by centrifugation at 2000 rpm for 15 min, and the supernatant was lyophilized to obtain *p*-toluene sulfonic acid-modified CS. The preparation of SNO-CS mainly involves the amide condensation reaction between the amino group on CS and the carboxyl group on SNO. First, 1 mL SNO (1 mol mL^-1^), 170.5 mg EDC, and 126.5 mg NHS were mixed in 10 mL DMF, then stirred in an ice bath for 30 min before adding 50 mg *p*-toluene sulfonic acid-modified CS and stirring for another 20 h in 0 ℃. The resulting mixture was then rinsed three times with methanol, freeze-dried, and stored at -20 °C. The SNO-CS structure was characterized using an FT-IR spectrometer (ALPHAII, Bruker) and a UV-visible photometer. Different amounts of SNO (concentrations ranging from 25, 50, 75, 100, 125.0, and 166.7 μmol L^-1^) and a condensation agent were added to quantify the grafting amount of SNO in DMF and stirred for 30 min. Equal amounts of *p*-toluene sulfonic acid-modified CS were dissolved in DMF and reacted in an ice bath for 20 h to conduct the amidation reaction. The concentration and standard fitting curves of SNO were obtained using the characteristic peak of the SNO group in the UV-Vis spectrum at 358 nm. Based on the fitting curve, the grafting efficiency of SNO on CS can be calculated, and the grafting ratio can be determined.

To prepare SNO-CS@MoS_2_, SNO-CS was first re-dispersed in deionized water at a concentration of 3 mg mL^-1^, and then gradually added into the MoS_2_ nanosheet dispersion (1.0 mg mL^-1^). The reaction mixture was kept in an ice bath for 4 h. Afterwards, excess SNO-CS in the supernatant was removed. The precipitate was collected and dried in a vacuum drying oven to obtain a black powdery product. CS@MoS_2_ was prepared using a parallel method. The structures of the SNO-CS@MoS_2_ and CS@MoS_2_ nanosheets were characterized using a UV-visible photometer and FT-IR spectrometer. The surface morphology of the SNO-CS@MoS_2_ nanosheets was observed using SEM and TEM. The particle size of SNO-CS@MoS_2_ and CS@MoS_2_, as well as the zeta potential of SNO-CS@MoS_2_, CS@MoS_2_, SNO-CS, and CS was analyzed using a Malvern particle size analyzer.

### In vitro photothermal effect of SNO-CS@MoS_2_

To evaluate the in vitro photothermal performance, SNO-CS@MoS_2_ (200 μg mL^-1^) was placed in a 2 mL plastic centrifuge tube, and then irradiated using an 808 NIR laser at 0.50, 0.75, or 1.00 W cm^-2^ for 10 min. The temperature values and thermal images were recorded at 15 s intervals using an infrared thermal imager camera (FLIR ONE Pro, TELEDYNE FLIR). Similarly, the dependence of photothermal performance on concentration was also examined. To calculate the photothermal conversion efficiency (*η*) [40], ultra-pure water and 200 μg mL^-1^ of SNO-CS@MoS_2_ were first irradiated using an 808 NIR laser at 1.0 W cm^-2^ for 15 min, then cooled down naturally for another 20 min. Finally, η was calculated using the following equation:

1 $$\eta =\frac{hs\left({T}_{max}-{T}_{amb}\right)-{Q}_{0}}{I\left(1-{10}^{{-A}_{808}}\right)}$$

2 $$hs=\frac{m{C}_{water}}{{\tau }_{s}}$$

3 $${\tau }_{s}=-\frac{t}{ln\theta }$$

4 $$\theta =\frac{T-{T}_{amb}}{{T}_{max}-{T}_{amb}}$$

5 $${Q}_{0}=hs\left({T}_{water}-{T}_{amb}\right)$$

where *hs* can be calculated using Eq. (2), where *m* is the mass of the SNO-CS@MoS_2_ solution (g), *C*_*water*_ is the heat capacity of water (J/g·°C), and *τ*_*s*_ is the sample system time constant (s). *τ*_*s*_ can be calculated according to formula (3–4); in formula (3), *θ* is the dimensionless driving force, and t is the time. Moreover, in formula (4), *T*_*max*_ represents the maximum steady-state temperature of the mixed solution, *T*_*water*_ represents the maximum steady-state temperature of the water, and *T*_*amb*_ represents the room temperature. *Q*_*0*_ represents the background energy input without a photothermal agent and is calculated using Eq. (5).

To evaluate the photothermal stability of SNO-CS@MoS_2_, freshly synthesized MoS_2_, CS@MoS_2,_ or SNO-CS@MoS_2_ dispersed in PBS were kept at 4 °C for five days. Then, they underwent continuous irradiation and cooling for three cycles using an 808 nm NIR laser to assess the photothermal stability of these materials.

### In vitro NO release profiles of SNO-CS@MoS_2_

The NO release profiles of SNO-CS@MoS_2_ were detected using a Griess reagent kit. Briefly, different concentrations of SNO-CS@MoS_2_ (100.0, 150.0, and 200.0 μg mL^-1^ in PBS) were placed in a 2 mL of the plastic centrifuge tube and then irradiated using an 808 NIR laser at an intensity of 1.0 W cm^-2^ for 10 min. At predetermined intervals, 100 μL of the solution was removed and mixed with 50 μL Griess reagent. After an additional half-hour of incubation at 37 °C, the amount of NO produced was determined by measuring the absorbance of the mixture at 540 nm using a microplate reader (SpectroMAX M5, Molecular Devices). To further investigate the self-release behavior of SNO-CS@MoS_2_ in the application scenarios, where SNO-CS@MoS_2_ releases a large amount of NO in response to photothermal action and exerts efficient antibacterial effects, then residual SNO-CS@MoS_2_ slowly produces trace amounts of NO under physiological conditions to promote wound healing, SNO-CS@MoS_2_ exposed to 10 min of NIR light irradiation was collected and then placed in an incubator at 37 °C. At regular intervals, 50 μL of the mixture was extracted to determine the amount of NO produced.

### In vitro antibacterial property

The bacterial strains used in this study included *S. aureus*, methicillin-resistant *S. aureus (MRSA), E. coli* and extended-spectrum beta-lactamase (ESBL)-producing *E. coli*. Bacterial single colonies were transferred to tryptone soy broth (TSB) liquid culture medium and incubated overnight at 37 °C for 12 h. The bacteria were grown to the logarithmic stage, collected, washed, and then diluted with sterile PBS to OD600 = 0.1 for subsequent antibacterial experiments. To evaluate the antibacterial effect of SNO-CS@MoS_2_, 500 μL of bacteria was first incubated with different volumes of SNO-CS@MoS_2_ (1 mg mL^-1^) at 37 °C for 30 min, and then illuminated with an 808 nm laser at 1 W cm^-2^ density for 10 min. The bacterial suspension was then gradually diluted stepwise from 10^6^ to 10^1^ and uniformly inoculated onto TSB plates. After incubation at 37 °C for 18 h, the number of bacterial colonies was counted to evaluate the antibacterial effect. The bacterial survival rate is expressed as log_10_ CFU mL^-1^. Bacteria treated with PBS, MoS_2_ (100, 200, or 400 μg mL^-1^), or CS@MoS_2_ (100, 200, or 400 μg mL^-1^) served as controls. SNO-CS@MoS_2_ (100, 200, or 400 μg mL^-1^) was used as a control without NIR light irradiation.

Live/dead staining was performed to further evaluate the antibacterial activity of SNO-CS@MoS_2_. SYTO-9 and PI were used to distinguish viable and nonviable bacterial cells. Briefly, 200 μL SNO-CS@MoS_2_ (1 mg mL^-1^) was mixed with 800 μL of bacterial suspension (10^8^ CFU mL^-1^) in an eppendorf tube and at 37 °C for 30 min. Then, the bacteria suspension was irradiated as described above. The bacteria were then collected by centrifugation and stained with 100 μL of live/dead backlight bacterial viability kit for 30 min in the dark. Finally, the stained bacteria were washed twice with PBS and observed under a confocal fluorescence microscope (A1, Nikon) at 60× magnification to capture fluorescent staining images. SEM was used to observe changes in bacterial morphology. The bacteria were treated as described above, then fixed with glutaraldehyde at room temperature for 4 h. After fixation, the bacteria were washed thrice with 0.85% NaCl solution and dehydrated in an increasing gradient of ethanol concentrations (20%, 40%, 60%, 80%, 90%, and 100%) in sequence for 15 min. The bacteria were then sputtered with gold before SEM examination. To quantify protein leakage from bacteria following SNO-CS@MoS_2_ treatment, the bacteria were first treated as described above. The supernatant was collected to measure protein concentration using an enhanced BCA protein assay kit and a microplate reader at 560 nm. Variations in intracellular ATP levels in response to the SNO-CS@MoS_2_ therapy were evaluated using an improved ATP assay kit. The bacteria were initially treated as described previously, collected, and lysed with 200 μL lysis buffer. Finally, the supernatant was collected and quantified using a luminance-mode microplate reader.

### Cell cultivation and in vitro cytotoxicity assay

L929 cells were cultured in Dulbecco’s modified Eagle’s medium (DMEM, Gibco) containing 10% (v/v) fetal bovine serum (FBS, Gibco) and 1% (v/v) penicillin-streptomycin (P&S, Gibco) at 37 ℃, 5% CO_2_. HUVECs were cultured in endothelial cell medium (ECM, Sciencell) containing 5% (v/v) FBS, 1% (v/v) growth factor (ECGS/ECGF), and 1% (v/v) P&S at 37 ℃, 5% CO_2_.

The cytotoxicity of SNO-CS@MoS_2_ against L929 cells was assessed using the MTT assay. L929 cells were seeded at a density of 5 × 10^3^ cells per well in 96-well plates overnight and then treated with SNO-CS@MoS_2_ at 10, 20, 50, 100, 200, and 500 μg mL^-1^ doses for another 24 h. Next, 10 μL of MTT reagent was added to each well and incubated for another 4 h. After carefully removing the supernatant from each well, 150 μL of dimethyl sulfoxide (DMSO) was added, and the absorbance of each well at 570 nm was measured using a microplate reader. The live/dead cell-staining assay was performed as follows. Briefly, after 1, 3, or 5 days of incubation with SNO-CS@MoS_2_ (200 μg mL^-1^), L929 cells were first stained with calcein-AM and PI double fluorescent stain for 30 min in the dark and then imaged using an inverted fluorescence microscope (Axio Observer 3 materials, ZEISS). Cells treated with PBS, SNO-CS (200 μg mL^-1^), and MoS_2_ (200 μg mL^-1^) served as controls.

### Hemolysis assay

Whole blood was extracted from healthy rat hearts by using an anticoagulant. To prepare red blood cell suspensions (RBCs), whole blood was first centrifuged, and the obtained RBCs were then redispersed in PBS. To evaluate the hemolysis rate of SNO-CS@MoS_2_, 1 mL of SNO-CS@MoS_2_ at concentrations ranging from 10 to 500 μg mL^-1^ was first mixed with 20 μL of RBCs and incubated at 37 °C for 4 h. Following centrifugation at 3000 rpm for 15 min, 100 μL of the supernatant was transferred to a 96-well plate, and the absorbance of each well was measured at 542 nm using a spectrophotometric microplate reader. RBCs treated with SNO-CS and MoS_2_ (50–400 μg mL^-1^) served as the control, PBS was used as a negative control, and double-distilled water was used as a positive control.

### Intracellular NO release

The intracellular NO generation behavior of SNO-CS@MoS_2_ in the absence of NIR irradiation was assessed as follows. Briefly, HUVECs were inoculated onto 96-well plates at a density of 1 × 10^3^ cells per well overnight. Then, 20 μL of SNO-CS-@MoS_2_ (1 mg mL^-1^) was added, and the cells were cultured for another 6 h. The cells were then exposed to 100 μL of DAF-FM DA (1 × 10^− 3^ M) for 30 min in the dark. Finally, cells were washed twice with PBS and observed under an inverted fluorescence microscope. Cells treated with PBS, SNO-CS (30 μg mL^-1^), and MoS_2_ (200 μg mL^-1^) served as the control, SNO-CS, and MoS_2_ groups, respectively.

### Cell scratching healing

Cell scratch experiments were performed to assess the effect of SNO-CS@MoS_2_ on promoting wound healing. Briefly, L929 cells were seeded at 1.5 × 10^5^ cells per well into 12-well plates and cultured to 80% confluence. The cell monolayer was then scraped with a sterile pipette tip (200 μL) and washed three times with PBS. Subsequently, the cells were incubated with fresh serum-free medium containing SNO-CS@MoS_2_ (200 μg mL^-1^) for 6, 18, or 24 h. After that, the cells were stained with crystal violet and observed under an inverted microscope. Cells treated with PBS, SNO-CS (30 μg mL^-1^), and MoS_2_ (200 μg mL^-1^) served as control, SNO-CS and MoS_2_ groups.

### Angiogenesis assay

To assess the angiogenic potential of SNO-CS@MoS_2_, a tube formation assay was performed. Briefly, HUVECs (3.5 × 10^3^) were seeded overnight in 48-well plates, then exposed to SNO-CS@MoS_2_ (200 μg mL^-1^)-containing molecular cellular and developmental biology 131 (MCDB131, pricella) medium (containing 2% (v/v) FBS and 1% (v/v) P&S) for 12 h. Afterward, the cells were digested and reseeded in 48-well plates pre-spread with matrix gel for 3 h. The cells were then stained with calcein fluorescent dye and observed using an inverted fluorescence microscope. The total length and the number of junctions were assessed by using the Image J software with an angiogenesis analyser plugin. Cells treated with PBS, SNO-CS (30 μg mL^-1^), and MoS_2_ (200 μg mL^-1^) served as the control, SNO-CS, and MoS_2_ groups, respectively. Besides, HUVECs were treated as described above. The cells were then collected to measure the mRNA and protein expression levels of CD31 and VEGF by qRT-PCR and western blotting. The primer sequences for angiogenesis-related genes (*CD31* and *VEGFα*) are listed in Table S1.

### In vivo treatment of S. aureus-infected full-thickness cutaneous wound model

All studies involving animals were reported following the ARRIVE guidelines and conducted according to National Research Council guidelines for the care and use of laboratory animals. Sprague-Dawley rats (eight weeks, Male) were obtained from Biotech Co. Ltd. (Beijing, China) and acclimatized for one week prior to the study. To establish the *S. aureus*-infected full-thickness cutaneous wound model, the rats anesthetized with an intramuscular injection of ketamine (100 mg mL^-1^) and xylazine (20 mg mL^-1^) at 1 mL kg^-1^ of body weight. The back of rats were shaved, and a round full-thickness cutaneous wound (10 × 10 mm) area was created using a punch, followed by inoculation with 10 μL of *S. aureus* suspension (10^8^ CFU mL^-1^) into the wound. Subsequently, all rats were randomly divided into five groups (n = 8): (1) PBS + NIR, (2) SNO-CS + NIR, (3) MoS_2_ + NIR, (4) SNO-CS@MoS_2_, and (5) SNO-CS@MoS_2_ + NIR. The wound area was treated by direct injection of 200 μL of PBS or the materials three times during the entire treatment period. Rats in NIR light irradiation group were irradiated with an 808 nm NIR laser at 1 W cm^-2^ for 5 min. Thermal images were acquired by an infrared thermal imager every 50 s. On day 1, the antibacterial activity in vivo was measured by swabbing the infected wound for 10 s and counting the colonies in PBS. The wound area was photographed using a digital camera on days 0, 3, 7, 9, and 11 to determine the wound closure rate (WCR). The WCR was calculated using the following equation, where *S*_*0*_ represents the wound area on day 0, and *S* represents the wound area on the photographed day.

$$WCR \left(\%\right)=\frac{\left({S}_{0}-S\right)}{{S}_{0}} \times 100\%$$

At the end of treatment, the rats were sacrificed, and blood was collected using a standard vein blood collection technique for hematology analysis. The safety of the treatment process was assessed by comparing the number of red blood cells (RBC), white blood cells (WBC), hemoglobin (HGB), hematocrit (HCT), alkaline phosphatase (ALP) and aspartate aminotransferase (AST). The wound tissues and viral organs were fixed, embedded, sectioned, and stained with hematoxylin and eosin (H&E), Masson’s trichrome, and anti-CD31 antibodies. All sections were observed under an inverted fluorescence microscope.

### Statistical analysis

The normal distribution of the data was verified using the Kolmogorov-Smirnov test. Significant differences in these variables were detected using a one-way analysis of variance (ANOVA). Statistical significance was set at P < 0.05.

### Results and discussion

#### Preparation and characterization of SNO-CS@MoS_2_

The preparation of SNO-CS@MoS_2_ mainly includes three steps (Scheme 1 A): 1) exfoliation of bulk MoS_2_ into mono- and few-layer 2D nanosheets of MoS_2_, (2) preparation of SNO-modified CS (SNO-CS), and (3) electrostatic adsorption of SNO-CS on the surface of MoS_2_ nanosheets. To prepare mono- and few-layer MoS_2_, TBAOH was employed as the intercalation agent to reduce the van der Waals interactions between the bulk MoS_2_ layers [41]. Subsequently, high-frequency ultrasound was employed to strip multiple layers of bulk MoS_2_ into mono- and few-layer MoS_2_ through acoustic cavitation [42]. The resulting mono- and few-layer MoS_2_ were then redispersed in deionized water, resulting in a homogenous and transparent dark green suspension. The successful preparation of mono- and few-layer MoS_2_ was confirmed by UV-vis absorption spectroscopy, morphological characterization, and Raman spectroscopy.

**Fig. 1:**
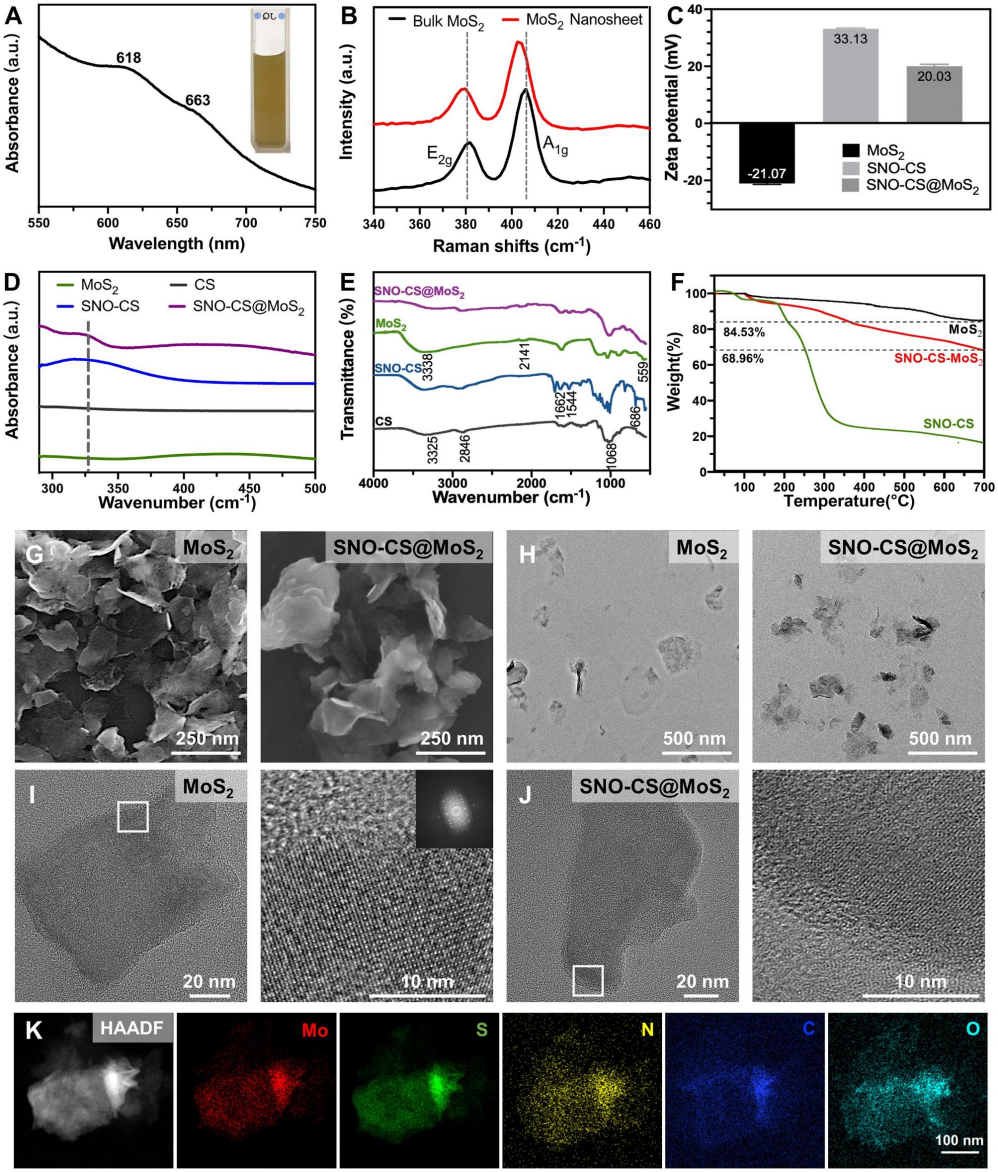
Spectroscopic characterization of SNO-CS@MoS_2_ nanosheets. (**A**) UV-vis absorbance spectra of MoS_2_ nanosheets. (**B**) Raman spectra of bulk MoS_2_ and MoS_2_ nanosheets. (**C**) Zeta potential of SNO-CS, MoS_2_ nanosheets and SNO-CS@MoS_2_ nanosheets. **D**-**E**) FT-IR spectra and UV-vis spectra of CS, SNO-CS, MoS_2_ nanosheets and SNO-CS@MoS_2_ nanosheets. **F**) TGA curves of MoS_2_ nanosheets, SNO-CS and SNO-CS@MoS_2_ nanosheets. **G**-**H**) SEM and TEM of MoS_2_ nanosheets and SNO-CS@MoS_2_ nanosheets. **I**-**J**) High-resolution TEM images of MoS_2_ nanosheets and SNO-CS@MoS_2_ nanosheets. **K**) Elemental mappings of Mo, S, C, N and O of SNO-CS@MoS_2_ nanosheets

Figure 1 A displays the UV-vis absorption spectrum of the MoS_2_ nanosheet, depicting characteristic absorption bands at 663 and 618 nm, which arise from the direct excitonic transitions at the K point of the Brillouin zone [43]. The presence of bands at 666 and 608 nm confirms the existence of mono- and few-layer MoS_2_, indicating the successful exfoliation of bulk MoS_2_ into nanosheets [44]. The microstructure and morphology of MoS_2_ nanosheets were characterized by SEM and TEM as shown in Fig. 1G-H, revealing a single or few-layered sheet-like structure with a smooth surface. Figure 1B displays Raman spectra of bulk MoS_2_ and MoS_2_ nanosheets, which reveal the characteristic peaks of MoS_2_: E^1^_2g_ and A_1g_, where the E^1^_2g_ peak is the in-plane bending mode, and the A_1g_ peak is the out-of-plane phonon mode related mostly to the stretching of the sulfur atoms [45]. It has been reported that the E^1^_2g_ and A_1g_ peaks in mono- and few-layer MoS_2_ typically exhibit a redshift and blueshift, respectively, along with a decrease in intensity compared to that of bulk MoS_2_ [44]. Notably, our sample exhibited a similar trend, as evidenced by the E^1^_2g_ and A_1g_ peaks of bulk MoS_2_ at 381 and 406 cm^-1^, respectively. In contrast, the E^1^_2g_ and A_1g_ peaks of the resulting MoS_2_ nanosheets shifted to 380 and 403 cm^-1^, respectively, and the intensities of the two peaks were reduced, confirming the successful preparation of mono- and few-layer MoS_2_.

The preparation of SNO-CS involves amide condensation reactions between the carboxyl groups on SNO and the amino groups on CS (Fig. S1). The UV-vis absorption and FT-IR spectra of SNO-CS confirmed the successful synthesis of SNO-CS. It has been reported that the S-NO group has a characteristic absorption band at 335–338 cm^-1^ in UV-vis spectrum, which is attributed to the allowed n_0_→ π* transition [46]. The characteristic absorption band of the S-NO group can be observed at 330–350 cm-1 in the UV-vis spectrum of SNO-CS (Fig. 1D). The synthesis of SNO-CS was further verified by FT-IR spectra in Fig. 1E. SNO-CS exhibits absorption spectra similar to pure CS [47]. Furthermore, in the spectra of SNO-CS, the characteristic bands found at 1516 and 1634 cm^-1^ correspond to the I and II bonds of C = O, respectively. The peak representing the N-H bond is also observed in the range of 3500–3700 cm^-1^, which together prove the amide bond existence [48]. Furthermore, SNO-CS also demonstrated a new peak at 686 cm^-1^ corresponding to the -S-N = bond [30]. The above analysis proves the successful combination of SNO-CS. Furthermore, the grafting rates of 3-(nitroso) propionic acid on CS were calculated using the UV-vis spectra and a standard curve of 3-(nitroso) propionic acid (Fig. S2A–B). The grafting rates were concentration-dependent, and at a concentration of 125 μmol L^-1^, the grafting efficiency of 3-(nitroso) propionic acid was 10.31%, resulting in a maximum grafting ratio of 15.46% (Fig. S2C). The above SNO-CS were used for the subsequent synthesis of SNO-CS@MoS_2_.

Through electrostatic interactions, we synthesized SNO-CS-coated MoS_2_ (SNO-CS@MoS_2_) following the successful creation of mono- and few-layer MoS_2_ and SNO-CS. The successful preparation of SNO-CS@MoS_2_ was confirmed by the following results:1) zeta potential and particle size distribution, 2) morphological characterization, 3) elemental mapping, 4) UV-vis absorption spectroscopy, and 5) FT-IR spectra. In detail, the zeta potential value charged from − 21.07 to 20.03 mV (Fig. 1C), and the average size increased from 127.5 to 341.1 nm (Fig. S3A-B) after coating of SNO-CS on MoS_2_. Additionally, the microstructures of MoS_2_ and SNO-CS@MoS_2_ were characterized by SEM and TEM in Fig. 1G-H. Compared to pure MoS_2,_ SNO-CS@MoS_2_ exhibited thicker and more uniformly dispersed nanostructures with a rough and vague surface. The above experiments proved that the combination of SNO-CS and MoS_2_ resulted in significant changes in the surface morphology and roughness of the original MoS_2_. Through measurements of TEM images, the average size of pure MoS_2_ is approximately 120 nm, which is consistent with the results obtained from DLS measurements. However, the average size of SNO-CS@MoS_2_ is approximately 260 nm, which is smaller than the results obtained from DLS measurements. The size difference of the SNO-CS@MoS_2_ measured by TEM and DLS was mainly attributed to DLS presenting wet samples. High-resolution TEM was further used to analyze the structure. The TEM images showed a clear lattice structure of MoS_2_ (Fig. 1I).The clarity of the lattice structure in SNO-CS@MoS_2_ is reduced due to the adsorption of SNO-CS (Fig. 1J). Elemental mapping was conducted to determine the elemental composition of SNO-CS@MoS_2_ in Fig. 1K and Table S2. The results confirmed the presence of Mo, S, C, N, and O, further confirming SNO-CS loading. The above results were further verified by the UV-vis absorption spectra in Fig. 1D and FT-IR spectra in Fig. 1E. The UV-vis absorption spectra of SNO-CS@MoS_2_ showed a shift in the absorption band of pure MoS_2_ from 450 to 437 cm^-1^ after SNO-CS modification. The characteristic absorption band of the S-NO group at 330–350 cm^-1^ was also observed. Moreover, UV-vis-NIR absorbance spectra of MoS_2_ showed strong UV to NIR absorbance, which was not affected by the surface SNO-CS modification (Fig. S4). The mass extinction coefficient of MoS_2_ nanosheets at 800 nm was calculated to be 7.45 L g^-1^ cm^-1^, similar to values measured by other researchers [49]. In addition, FT-IR spectra clearly demonstrated that SNO-CS@MoS_2_ had absorption spectra similar to those of SNO-CS and pure MoS_2_. All the above results prove the successful synthesis of SNO-CS@MoS_2_. Furthermore, the loading amount of SNO-CS on MoS_2_ was estimated using thermogravimetric analysis (TGA), as demonstrated in Fig. 1F. The TGA curve of MoS_2_ shows significant weight loss between 400 and 500 °C due to the oxidation of MoS_2_ to MoO_3_. The thermal decomposition profile of SNO-CS@MoS_2_ is like SNO-CS, it gradually decreases between 250 and 600 ºC, and the weight loss below 150 °C can be attributed to moisture vaporization. According to TGA data, it was estimated that ~ 14% weight of SNO-CS was loaded onto the MoS_2_ nanosheets.

Furthermore, to demonstrate that the synergism of SNO-CS on the PTT antibacterial activity of MoS_2_ mainly originates from NO, we synthesized CS@MoS_2_ using similar methods. In addition, the successful preparation of CS@MoS_2_ was confirmed by particle size and zeta potential analysis, as displayed in Fig. S3C-D. CS@MoS_2_ presents a positively charged nanostructure with an average size of 212 nm. Long-term stability tests were conducted on MoS_2_, CS@MoS_2,_ and SNO-CS@MoS_2_. Fig. S5A-B depict photographs of the solution color and particle size change at different time points (day 1, 3, and 5) for freshly synthesized MoS_2_, CS@MoS_2,_ and SNO-CS@MoS_2_ in the PBS solution. MoS_2_, CS@MoS_2,_ and SNO-CS@MoS_2_ exhibited good dispersibility on day 1. However, MoS_2_ aggregated on day 3, and the average particle size increased to approximately 1 μm. In contrast, CS@MoS_2_ and SNO-CS@MoS_2_ exhibited high dispersion and stability on day 5 (Fig. S5C). This experiment demonstrated that the interlayer re-stacking of MoS_2_ can be inhibited after CS or SNO-CS modification, and SNO-CS@MoS_2_ exhibits superior stability and shows good prospects for application in vivo.

### Photothermal performance and NO generation behavior

**Fig. 2:**
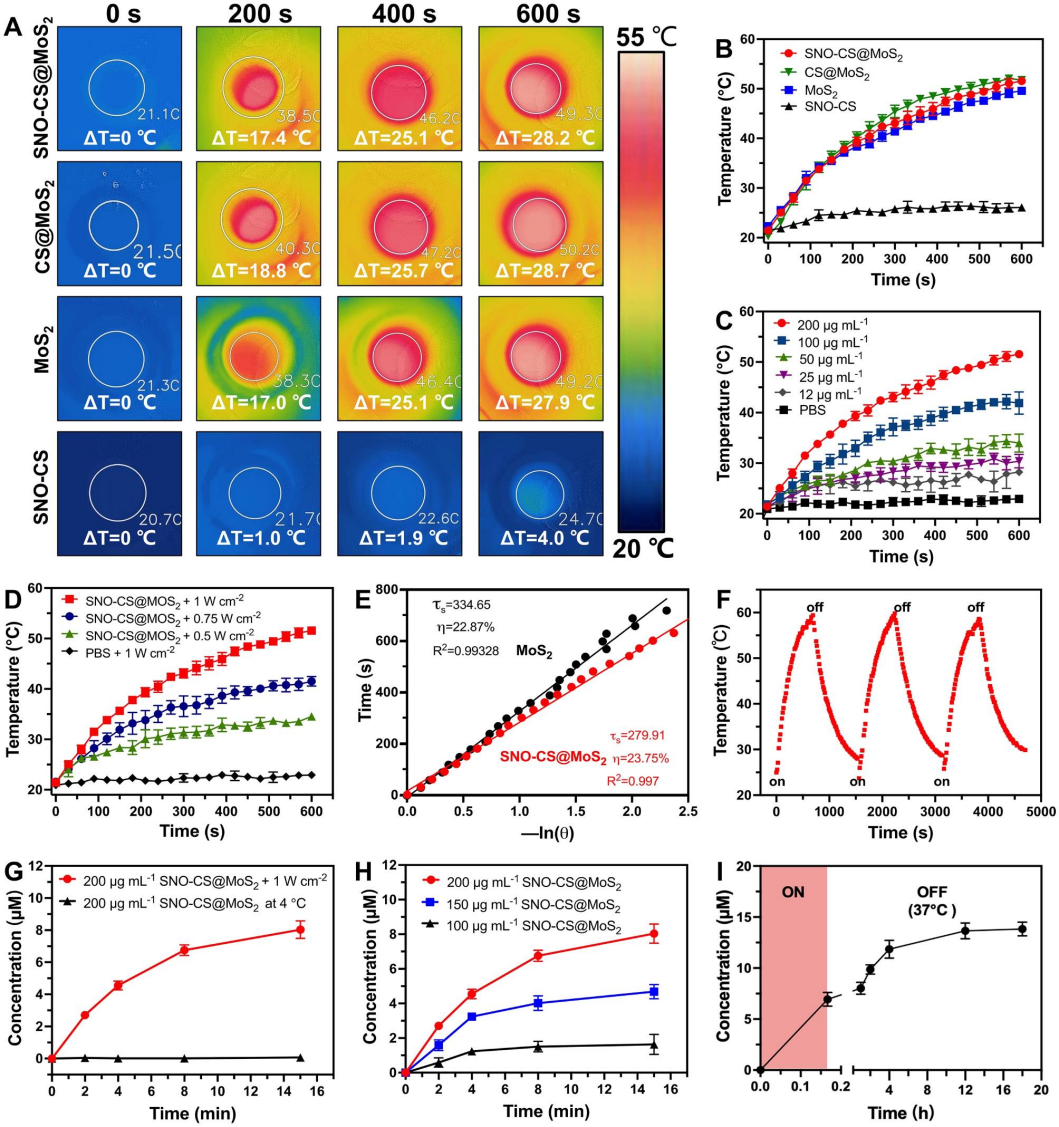
The photothermal effect and NO generation behavior of SNO-CS@MoS_2_ nanosheets. (**A**) Infrared thermal images of SNO-CS, MoS_2_, CS@MoS_2_ and SNO-CS@MoS_2_ (concentration: [MoS_2_] = 200 μg mL^-1^, [SNO-CS] = 30 μg mL^-1^) under NIR irradiation (808 nm, 1 W cm^-2^). and (**B**) corresponding temperature profiles. (**C**) Temperature profiles of SNO-CS@MoS_2_ in different concentration (12, 25, 50, 100 and 200 μg mL^-1^) under NIR irradiation (808 nm, 1 W cm^-2^). (**D**) Temperature profiles of SNO-CS@MoS_2_ at different light intensity under an 808 nm laser (0.5, 0.75 and 1 W cm^-2^). (**E**) The cooling time plot versus − ln(𝜃) of MoS_2_ and SNO-CS@MoS_2_ nanosheets (200 μg mL^-1^). (**F**) Temperature elevations of SNO-CS@MoS_2_ nanosheets for three NIR light irradiations cycles under an 808 nm laser (200 μg mL^-1^, 1 W cm^-2^). (**G**) NO release curves of SNO-CS@MoS_2_ nanosheets under NIR irradiation (808 nm, 1 W cm^-2^) or stored at 4 °C. (**H**) NO release curves of SNO-CS@MoS_2_ nanosheets in different concentration (100, 150 and 200 μg mL^-1^) under NIR irradiation (808 nm, 1 W cm^-2^). (**I**) Accumulation NO release within 18 h on SNO-CS@MoS_2_ nanosheets after 10 min of irradiation (808 nm, 1 W cm^-2^)

After successfully preparing SNO-CS@MoS_2_, we systematically evaluated the photothermal properties of SNO-CS@MoS_2_. Figure 2 A-B display the temperature increase of SNO-CS@MoS_2_, CS@MoS_2_, MoS_2,_ and SNO-CS under 808 nm NIR light irradiation. The results demonstrate that MoS_2_ has excellent photothermal conversion performance, and the coatings of CS and SNO-CS did not affect the photothermal performance of MoS_2_. Studies have demonstrated that temperatures over 50 °C kill bacteria by inactivating enzymes related to bacterial life activities [3]. The temperature of SNO-CS@MoS_2_ solution increased by 25 °C after 6 min of irradiation, indicating that it could achieve effective antibacterial performance in vivo. In addition, the results in Fig. 1C-D show that the photothermal properties of SNO-CS@MoS_2_ nanosheets depend on the concentration and laser intensity. Under a fixed laser power (1.0 W cm^-2^), the solution is heated with increasing concentrations. Moreover, the solution temperature increased with the laser intensity at a fixed concentration (200 μg mL^-1^). The photothermal conversion efficiency (PCE) of the freshly prepared nanomaterials is calculated in Fig. 2E and S6C. The PCE of SNO-CS@MoS_2_ was 23.75%, barely similar to that of CS@MoS_2_ (24.37%) and MoS_2_ (22.87%), and is related to the high photothermal stability of freshly prepared MoS_2_. The long-term photothermal stability of SNO-CS@MoS_2_ was also investigated. After five days of setting, three heating/cooling cycles under 808 nm radiation were applied to MoS_2_, CS@MoS_2,_ and SNO-CS@MoS_2_ (Fig. 2F and S6A-B). The temperature of pure MoS_2_ reached only 34 °C after 10 min of irradiation, which was much lower than that on the first day (49.57 °C). In contrast, CS@MoS_2_ and SNO-CS@MoS_2_ exhibit high photothermal stability across all three switching cycles. This result confirmed that the long-term photothermal stability of MoS_2_ nanosheets was greatly improved after chitosan or SNO-CS modification. In conclusion, the above investigations depicted that SNO-CS@MoS_2_ possesses excellent photothermal characteristics and is a promising photothermal agent for both in vivo and i*n vitro* applications.

After demonstrating the excellent photothermal performance of SNO-CS@MoS_2_, we further evaluated the NO-releasing behavior of SNO-CS@MoS_2_ using the Griess assay. Quantitative NO investigation was conducted using a standard curve of NO (Fig. S7). Figure 2G depicts that under 808 nm NIR irradiation (1 W cm^− 2^), the amount of NO generated by SNO-CS@MoS_2_ was much higher than that stored at 4 °C, indicating effective control of NO release by NIR laser irradiation. Furthermore, NO release from the SNO-CS@MoS_2_ nanosheets was concentration-dependent (Fig. 2H). The amount of released NO increased with higher concentrations. Among them, 200 μg mL^− 1^ of SNO-CS@MoS_2_ can release nearly 7 μmol L^− 1^ of NO after 8 min of NIR irradiation, fully meeting the effective antibacterial concentration requirements.

In addition to the efficient release of NO by laser irradiation, we further demonstrated that SNO-CS@MoS_2_ can produce low concentrations of NO in a physiological environment. To detect the NO release under the physiological environment, we placed RSNO, SNO-CS and SNO-CS@MoS_2_ in PBS under natural light at 37 ℃. The results showed that the NO release from the three groups was basically the same, indicating that the slow release of RSNO was mainly related to natural light and room temperature [50, 51] (Fig. S8). Furthermore, we collected the SNO-CS@MoS_2_ solution after 10 min of irradiation and monitored its long-term NO-release behavior at 37 °C. As illustrated in Fig. 2I, SNO-CS@MoS_2_ can release low concentrations of NO after the removal of NIR irradiation, and the increased amount of NO could be cumulative to 6.88 μmol L^− 1^ from 10 min to 18 h. In conclusion, the above experiments have demonstrated that SNO-CS@MoS_2_ nanosheets possess the ability to generate NO under photothermal control and exhibit sustained NO generation in a physiological environment.

### In vitro antibacterial effects of SNO-CS@MoS_2_

**Fig. 3:**
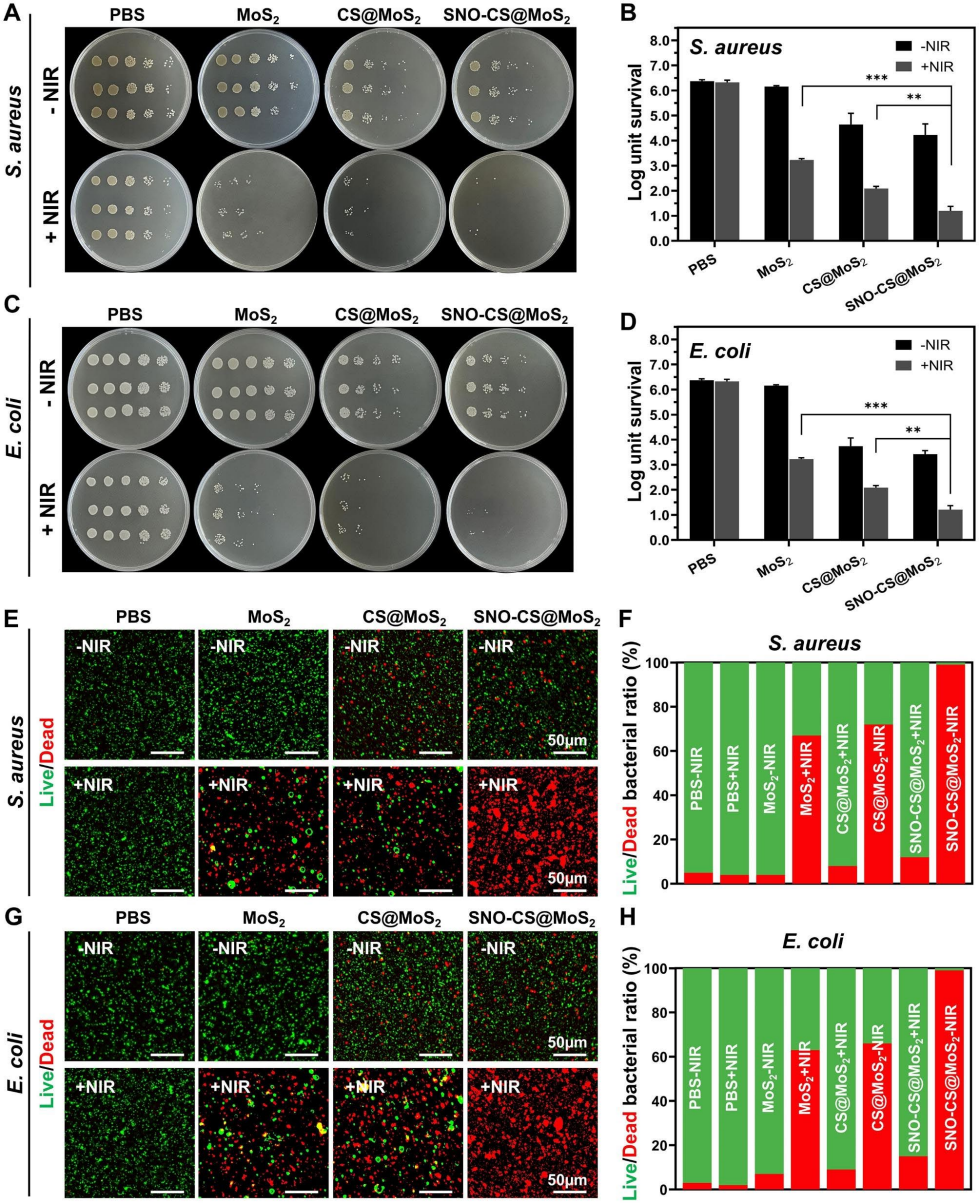
Antibacterial effect of SNO-CS@MoS_2_. (**A**) Photographs of bacterial colonies formed by *S. aureus* after various treatment; (**B**) The corresponding bacterial viabilities of *S. aureus*; (**C**) Photographs of bacterial colonies formed by *E. coli* after various treatment; (**D**) The corresponding bacterial viabilities of *E. coli*; **E**-**F**) Fluorescent images of *S. aureus* and corresponding statistical data; **G**-**H**) Fluorescent images of *E. coli* and corresponding statistical data. (Laser: 808 nm, 1.0 W cm^-2^, 10 min; Concentration: 200 μg mL^-1^)

The above studies have demonstrated the photothermal property and NO-producing capability of SNO-CS@MoS_2_, which prompted further investigation into its antibacterial properties. The antibacterial experiment against Gram-positive *S. aureus* and Gram-negative *E. coli* was conducted using the plate counting method. As displayed in Fig. 3A-D, in the absence of a NIR laser, the PBS and MoS_2_ treatment groups exhibited no antibacterial effect, whereas the CS@MoS_2_ and SNO-CS@MoS_2_ groups demonstrated some antibacterial effect, which may be attributed to the inherent antibacterial property of CS [52]. After 10 min of exposure to NIR irradiation, the survival rate of bacteria in the MoS_2_ and CS@MoS_2_ groups displayed a significant downward trend compared with the corresponding groups without irradiation treatment. The CS@MoS_2_ + NIR group exhibited log reductions of 3.29 for *S. aureus* and 1.92 for *E. coli*, indicating that the local hyperthermia produced by PTT of MoS_2_ exhibited high antibacterial effects. The SNO-CS@MoS_2_ + NIR exerted the greatest antibacterial effect. Furthermore, the bacterial colonies of *S. aureus* and *E. coli* significantly decreased by 4.07 and 4.71 log, respectively, demonstrating that a high concentration of NO significantly enhances the antibacterial effect of PTT. Moreover, the synergistic effect of NO on MoS_2_ PTT antibacterial therapy was concentration-dependent. As displayed in Fig. S9-10, the synergistic effect of SNO-CS@MoS_2_ was more obvious at low concentrations (100 μg mL^− 1^) while inconspicuous at high concentrations (400 μg mL^− 1^). Moreover, an increase in the concentration of SNO-CS@MoS_2_ led to higher PTT temperatures, posing a risk of tissue damage. Therefore, we selected 200 μg mL^− 1^ as the final concentration. The live/dead staining technique confirmed the synergistic antibacterial action. The proportion of living and dead bacteria can be directly observed in the fluorescence images (Fig. 3E-H). Only red fluorescent spots were observed in *S. aureus* and *E. coli* of the SNO-CS@MoS_2_ + NIR group, implying that SNO-CS@MoS_2_ treatment with NIR killed almost all bacteria. Intense green fluorescence was still observed in MoS_2_ + NIR and CS@MoS_2_ + NIR groups. This research further demonstrates the feasibility and efficacy of the synergistic antibacterial activity of NO and PTT. Furthermore, the main issue with antibacterial agents is bacterial multidrug resistance. We further conducted in vitro antibacterial experiments against resistant bacteria using plate counting method. The results are generally align with the findings mentioned above (Fig. S11).

**Fig. 4:**
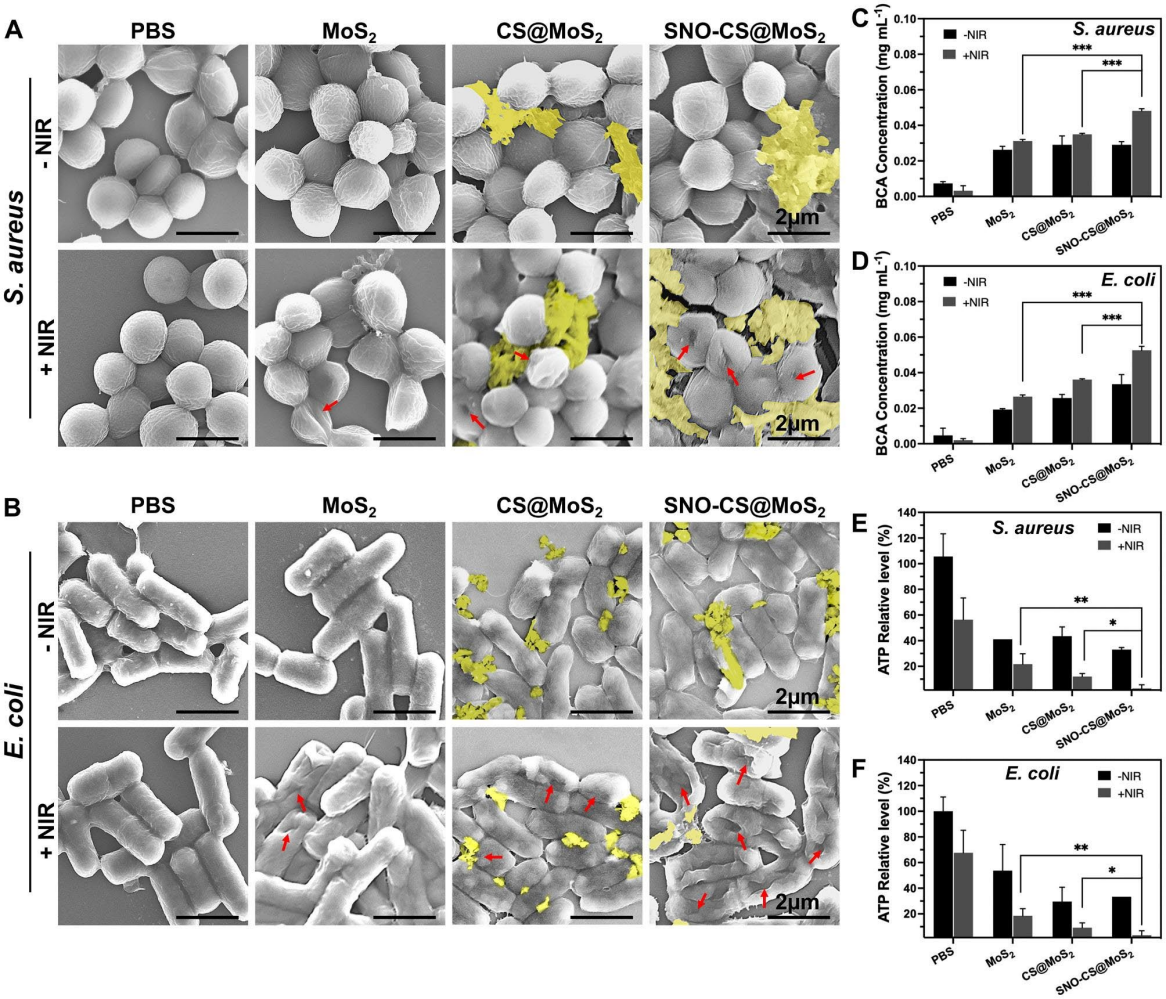
Antibacterial mechanisms of SNO-CS@MoS_2_ nanosheets. **A**-**B**) SEM images of *S. aureus* and *E. coli*. Red arrows marked the broken sites of bacteria. yellow mark covered the nanosheets. **C**-**D**) the amount of protein released from *S. aureus* and *E. coli* following receiving various treatments. **E**-**F**) Changes of intracellular ATP synthesis in *S. aureus* and *E. coli* after various treatments. (Laser: 808 nm, 1.0 W cm^-2^, 10 min; Concentration: 200 μg mL^-1^)

After confirming the excellent synergistic antibacterial performance of SNO-CS@MoS_2_, experiments were conducted to further investigate the antibacterial mechanisms. Firstly, changes in the morphology and membrane integrity of *S. aureus* and *E. coli* after SNO-CS@MoS_2_ treatment were observed using SEM. As demonstrated in Fig. 4A-B, in the presence or absence of NIR laser, both *S. aureus* and *E. coli* in the PBS treatment groups exhibited intact spherical morphology, characteristic rod-like structures, smooth surfaces, and intact cell membrane structures. Comparatively, MoS_2_ + NIR and CS@MoS_2_ + NIR caused varying degrees of membrane contraction and deformation. In contrast, treatment with SNO-CS@MoS_2_ + NIR resulted the most pronounced deformation and loss of cell integrity in *S. aureus* and *E. coli* (shown by red arrows), leading to bacteria death owing to the release of their contents. Moreover, the presence of nanomaterials attached to the bacterial surface (yellow overlay) was observed in the CS@MoS_2_ and SNO-CS@MoS_2_ groups. Based on these findings, we speculate that CS@MoS_2_ and SNO-CS@MoS_2_ can adsorbed onto bacteria through electrostatic interactions and damage the bacterial membrane by the physical effect of PTT and NO. The rupture of bacterial cell membranes further leads to massive protein leakage from the bacteria. As illustrated in Fig. 4C-D, the amount of protein released from *S. aureus* and *E. coli* in the SNO-CS@MoS_2_ + NIR group was approximately 1.4- and 1.5-fold higher than that in the other treatment groups, respectively. Severe breach of the bacterial cell membrane results in the loss of aerobic respiration-related enzymes on the cell membrane, thereby blocking intracellular ATP generation. The production of ATP of *S. aureus* and *E. coli* reduced by 97.19 and 96.57% after SNO-CS@MoS_2_ treatment with NIR irradiation (Fig. 4E-F). In conclusion, the above results indicate a significant synergistic antibacterial effect between PTT and NO. The potential antibacterial mechanism can be attributed to the following:1) the adhesion capability of the positively charged SNO-CS@MoS_2_; 2) the combination of the reactive byproducts of NO and physical damage from PTT, leading to the bacterial cell membrane rupture, substantial protein leakage and inhibition of intracellular ATP synthesis; and 3) NO byproducts may induce bacterial cell death through mechanisms such as DNA damage and protein dysfunction.

### Biocompatibility assay of SNO-CS@MoS_2_

**Fig. 5:**
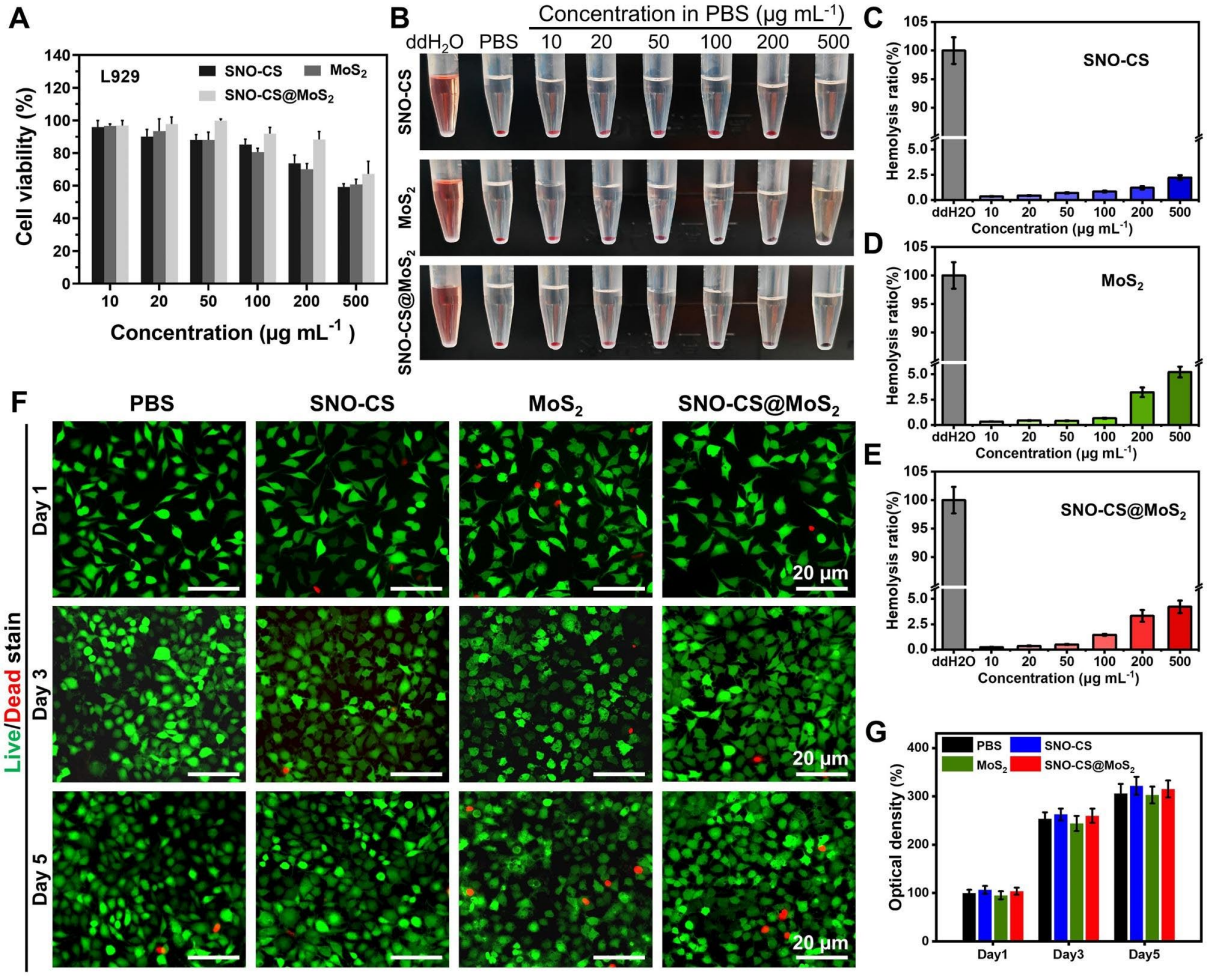
Biocompatibility and blood compatibility of SNO-CS@MoS_2_ nanosheets. **A**) MTT assay of different treatments in various concentration (10, 20, 50, 100, 200 and 500 μg mL^-1^). **B**-**E**) Hemolysis photographs and hemolysis ratio (%) of different treatments in various concentration (10, 20, 50, 100, 200 and 500 μg mL^-1^). **F**-**G**) Live/dead staining of L929 cells at 1, 3, and 5 d after treated with PBS, SNO-CS, MoS_2_, and SNO-CS@MoS_2_. (Concentration: [MoS_2_] = 200 μg mL^-1^, [SNO-CS] = 30 μg mL^-1^)

The biocompatibility of SNO-CS@MoS_2_ is essential for its subsequent biological applications. The cytotoxicity of SNO-CS@MoS_2_ in fibroblasts (L929 cells) was evaluated using the MTT assay and the hemolysis test. The biocompatibility after 24 h of co-incubation with different concentrations of nanomaterials was quantified in Fig. 5A by MTT assay. Below or equal to 200 μg mL^− 1^, over 85% of L929 cells survived after incubation with SNO-CS@MoS_2_, higher than MoS_2_ alone. This improvement in biocompatibility can be attributed to the encapsulation of SNO-CS. As demonstrated in Fig. 5B, insignificant hemolysis was observed in all treatment groups, and the quantification results depicted that the hemolysis rate was lower than 5% (Fig. 5C-E). Considering the antibacterial test results and biocompatibility findings, a concentration of 200 μg/mL of nanomaterials was chosen for subsequent experiments. To observe the long-term cellular biocompatibility of the materials, live/dead staining was used. Figure 5 F displays fluorescence images of live/dead staining of L929 cells after co-incubation with different nanomaterials for 1, 3, and 5 days. All treatment groups demonstrated low cytotoxicity, with only a few dead L929 cells (red fluorescence). Additionally, as the incubation period increased, the SNO-CS and SNO-CS@MoS_2_ groups exhibited a slightly more proliferative trend compared to the other groups.

### SNO-CS@MoS_2_ promoting angiogenesis and cell scratch healing

**Fig. 6:**
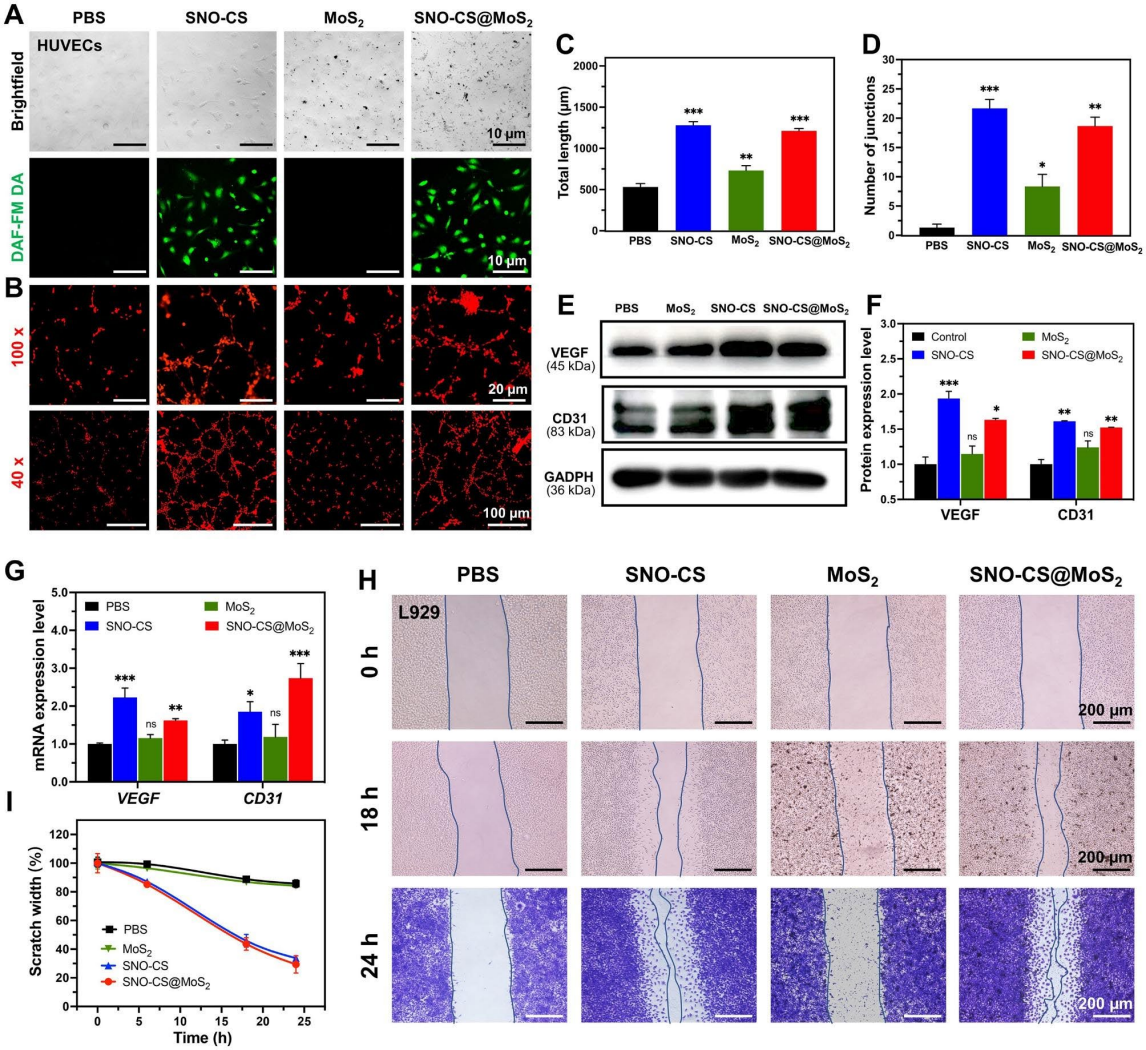
Mechanisms of SNO-CS@MoS_2_ promoting wound healing. (**A**) Brightfield and fluorescent image of HUVECs after treated with PBS, SNO-CS, MoS_2_, and SNO-CS@MoS_2_. DAF-FM DA used as NO probe; (**B**) Tube formation assay of HUVECs after various treatment; **C**-**D**) Quantitative analysis of the differences in the number of nodes and total length between groups. **E**-**F**) Protein expression levels and quantitative analysis of VEGF and CD31 in HUVECs after various treatment. **G**) *mRNA* expression levels of *VEGF* and *CD31* in HUVECs after various treatment. **H**-**I**) Images and quantitative analysis of cell migration of L929 cells after treated with PBS, SNO-CS, MoS_2_, and SNO-CS@MoS_2_. (Concentration: [MoS_2_] = 200 μg mL^-1^, [SNO-CS] = 30 μg mL^-1^)

In addition to efficiently killing bacteria in the infected wound area, promoting rapid wound healing is also a key aspect of effective wound care. The ability of low concentrations of NO to promote wound healing has been documented and proven to play an important role in promoting vascular regeneration and epithelial cell migration. First, DAF-FM DA was applied to track intracellular NO release. As displayed in Fig. 6A, after 12 h of co-culture, HUVECs in the SNO-CS and SNO-CS@MoS_2_ groups showed a strong green fluorescence signal than the PBS and MoS_2_ groups. It indicates that SNO-CS@MoS_2_ could gradually produce trace amounts of NO intracellularly under physiological conditions. Therefore, we evaluated its pro-angiogenic and pro-wound-healing properties. Figure 6B depicts that better tube formation was observed after 3 h of incubation with SNO-CS@MoS_2_ and SNO-CS, in sharp contrast to the PBS- and MoS_2_-treated groups. The number of nodes and the total length of the formed vessels were calculated in Fig. 6C–D using Image J software. These calculation parameters were substantially higher for the SNO-CS and SNO-CS@MoS_2_ groups compared to the PBS and MoS_2_ groups. In conclusion, the above results demonstrate that trace amounts of NO slowly released from SNO-CS@MoS_2_ can promote angiogenesis.

Vascular endothelial growth factor (VEGF) is a critical protein involved in angiogenesis. Currently, studies demonstrated that NO participates in the signal transduction process of VEGF-induced angiogenesis in vitro and in vivo [53]. Briefly, trace amounts of NO can mediate HIF-1 and HO-1 activation via the PI3K-Akt pathway, thereby upregulating VEGF expression [54]. Platelet endothelial cell adhesion molecule (CD31) is an important marker of vascular endothelial differentiation and is vital in vascular development by maintaining vascular function [55]. These indicators are commonly used to assess angiogenesis. Therefore, qRT-PCR and western blotting were used to confirm the angiogenic mechanism of SNO-CS@MoS_2_. Figure 6E-G depict VEGF and CD31 expression in HUVECs after co-culture with different materials for 24 h. The protein and *mRNA* expression levels of VEGF and CD31 were significantly higher in the SNO-CS and SNO-CS@MoS_2_ groups than in the PBS and MoS_2_ groups. These findings suggest that SNO-CS@MoS_2_ can efficiently release trace NO to activate VEGF and CD31 expression, thereby increasing vascular formation.

In the middle and final phases of wound healing, tissue reparative cells, such as epidermal cells and fibroblasts, migrate and proliferate towards the wound tissue to promote wound closure, which is a critical physiological response in the wound healing process. Studies have shown that NO is crucial for regulating wound epithelization and can promote the proliferation and migration of fibroblasts near the wound area [56]. Based on this, we next investigated the ability of NO to promote fibroblast migration using scratch experiments. As displayed in Fig. 6H-I, after the co-incubation for 24 h, the scratch healing rates of L929 cells in the SNO-CS and SNO-CS@MoS_2_ groups were noticeably higher compared to PBS and MoS_2_. The scratch healing rate of SNO-CS@MoS_2_ (70.64%) was much higher than PBS (14.25%). The results of the scratch experiment, along with the live/dead labeling of L929 cells, demonstrated that SNO-CS@MoS_2_ can enhance the migration and proliferation of L929 cells to the wound by producing trace amounts of NO at the infected wound site. In summary, SNO-CS@MoS_2_ can promote angiogenesis and the migration and proliferation of tissue repair cells to the wound by slowly producing a small amount of NO in the cells, thus significantly accelerating the infected wound healing.

### The performance of promoting wound healing in vivo

**Fig. 7:**
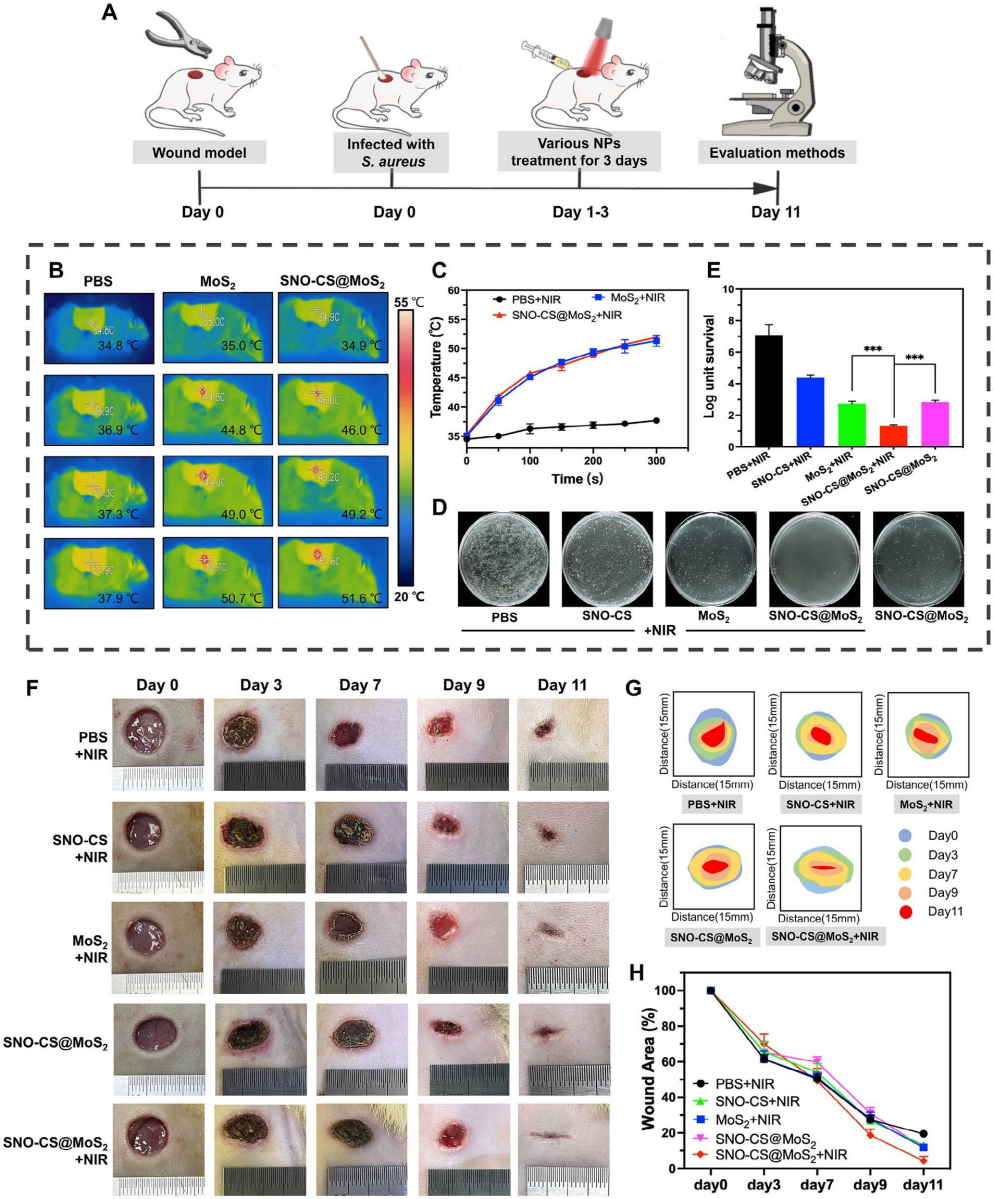
The efficiency of the SNO-CS@MoS_2_ on wound healing. **A**) Schematic illustration for the establishment of an infected wound model and the subsequent treatment regime. **B**-**C**) Thermal images of rats under NIR irradiation and the corresponding photothermal heating curves. **D**-**E**) Cultured bacteria colonies on plates separated from wound tissues after treatment of day 1 and relative survival log of bacteria. **F**) The Digital photos of wound closure in different treatment of PBS + NIR, SNO-CS + NIR, MoS_2_ + NIR, SNO-CS@MoS_2_ and SNO-CS@MoS_2_ + NIR on days 0, 3, 7, 9, and 11. **G**-**H**) Schematic diagram and quantification of the wound area after treatments on day 0, 3, 7, 9 and 11. (Laser: 808 nm, 1.0 W cm^− 2^, 10 min; Concentration: [MoS_2_] = 200 μg mL^− 1^, [SNO-CS] = 30 μg mL^− 1^)

Inspired by the excellent performance of SNO-CS@MoS_2_ in antibacterial, angiogenic, and rapid cell migration in vitro, a rat full-thickness skin defect model infected with S. aureus was developed to assess the efficacy of SNO-CS@MoS_2_ in infected wound healing in vivo. As illustrated in Fig. 7A, the infected wounds were treated with the materials and the corresponding NIR irradiation for the first 3 days of treatment. Figure 7B-C display the local changes in body temperature at the infected wound site during the first-day irradiation treatment. It can be observed that SNO-CS@MoS_2_ exhibited excellent photothermal conversion performance in vivo. The temperature in the wound area increased from 34.9 to 51.6 °C rapidly after 5 min of irradiation. To determine the ability to control NO generation in vivo, The solution from the wound area of the rat’s back was collected and the concentration of its NO was measured. The results are showed that SNO-CS@MoS_2_ + NIR could release nearly 3.54 μmol L^− 1^ of NO, while SNO-CS@MoS_2_ without NIR releases only 0.53 μmol L^− 1^ of NO (Fig. S12). The in vivo release of NO from SNO-CS@MoS_2_ was slightly lower than the above results of experiments in vitro (Fig. 2H). A more complex environment in the body may reduce the concentration of NO released due to wound exudate diluting the SNO-CS@MoS_2_ solution. In addition, behaviors such as movement and licking can affect the amount of material retained in the wound, thus reducing the amount of NO released. Although the concentration of NO released was reduced, it still met the effective antibacterial concentration. The in vivo antibacterial effect of SNO-CS@MoS_2_ is shown in Fig. 7D-E. The bacteria colony in wound tissue of SNO-CS@MoS_2_ + NIR group decreased from 6.58 to 1.52 log after the first-day treatment. Furthermore, the synergistic antibacterial effect of PTT and massive NO can further be proved by comparing with MoS_2_ + NIR and SNO-CS@MoS_2_ groups. This phenomenon is also supported by the anti-bacterial results of drug-resistant bacteria in vivo (Fig. S13). These results are consistent with the in vitro antibacterial assay.

Bacterial infection triggers an inflammatory response, including the infiltration of inflammatory cells and the release of inflammatory mediators. These inflammatory responses not only disrupt tissue structure but also inhibit cell proliferation and angiogenesis [57]. Killing bacteria effectively reduces the inflammatory response and promotes normal wound healing [58]. To evaluate the therapeutic efficacy of nanomaterials in wound healing, the wound healing process in each group was dynamically observed. SNO-CS@MoS_2_ treatment significantly accelerated infected wound healing, as verified by the results illustrated in Fig. 7F-H. The wound size of SNO-CS@MoS_2_ + NIR group decreased to 2.95% on day 11, while in the other groups, it remained higher than 10%.

**Fig. 8:**
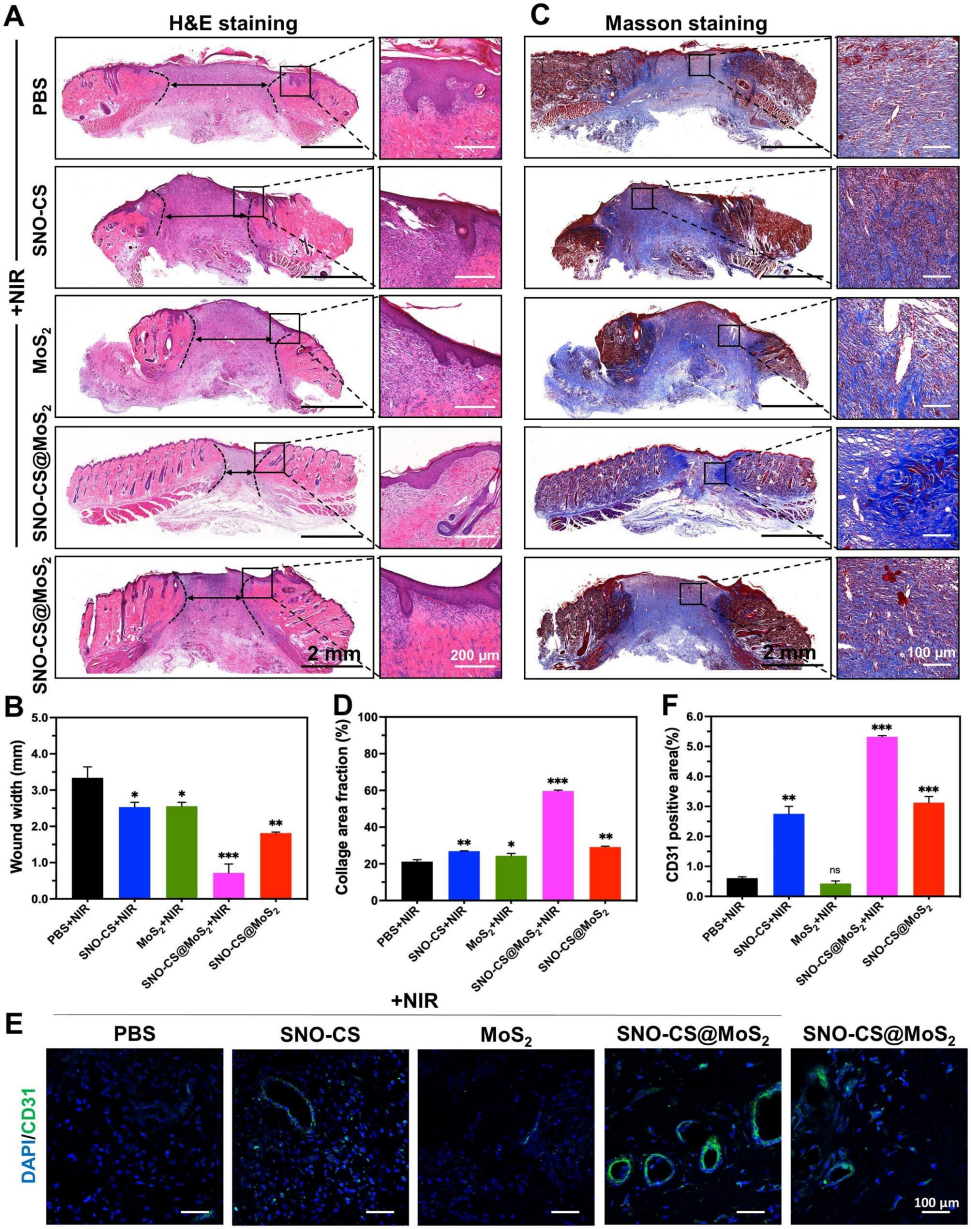
Histological analysis of the wound tissues. **A**-**B**) H&E staining and quantitative analysis of the tissues; **C**-**D**) Masson’s trichrome staining and quantitative analysis of the tissues; **E**-**F**) Immunofluorescence analysis (CD31) and quantitative analysis of the tissues

To further conduct a thorough assessment of the inflammatory reactions and the influence on blood vessel formation by SNO-CS@MoS_2_ in wound healing, skin wound tissues were collected from rats on day 11 for histological experiments. Figure 8 A and C display that SNO-CS@MoS_2_ + NIR treatment reduced inflammatory cell infiltration in the wound tissue and significantly promoted wound re-epithelialization. Compared to PBS and SNO-CS + NIR groups, the MoS_2_ + NIR and SNO-CS@MoS_2_ groups exhibited relatively intact neo-keratin, but large amounts of inflammatory cells were still infiltrating. Notably, SNO-CS@MoS_2_ + NIR treatment group showed not only a significant reduction in inflammatory cell infiltration, but also regular epidermal structures, well-proliferating fibroblasts, and even newborn skin attachments. Figure 8B and D display collagen deposition in the wound tissue after the different treatments. The SNO-CS@MoS_2_ + NIR group showed a higher level of collagen deposition compared to the other groups, with a new collagen area of approximately 60%. Additionally, the collagen fibers in this group were denser and more regularly arranged. These results further confirmed the ability of trace NO to promote the proliferation and migration of fibroblasts at the wound site. Furthermore, NO regulates endothelial cell functions such as proliferation, migration, and tube formation, essential for angiogenesis [59]. It also can modulate the expression of various angiogenic factors, including VEGF and fibroblast growth factor (FGF), influencing blood vessel formation [60]. CD31 was used for immunofluorescence staining to evaluate the angiogenesis-promoting effect of SNO-CS@MoS_2_. As expected, all NO-treated groups (SNO-CS + NIR, SNO-CS@MoS_2_, and SNO-CS@MoS_2_ + NIR) exhibited varying degrees of angiogenesis (Fig. 8E-F). Specifically, the SNO-CS@MoS_2_ + NIR group showed the highest expression of CD31, resulting from the slow release of NO, which considerably enhance vascular regeneration.

**Fig. 9:**
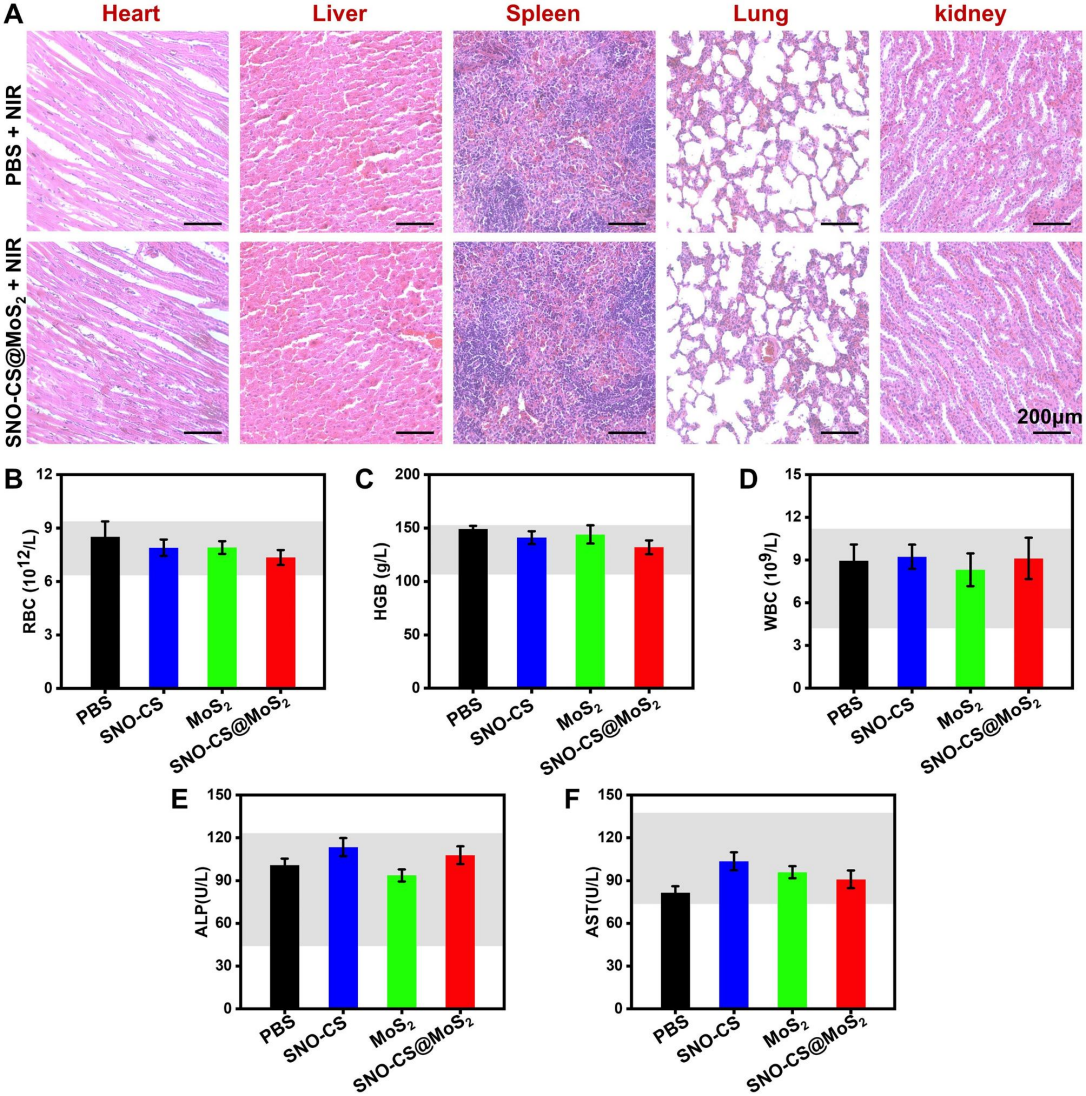
In vivo biocompatibility of SNO-CS@MoS_2_. (**A**) H&E staining of heart, liver, spleen, lung, and kidney of PBS + NIR and SNO-CS@MoS_2_ + NIR groups. The routine blood analysis for each treatment group on the 11th day, (**B**) white blood cell count (WBC), (**C**) hemoglobin (HGB), (**D**) red blood cell count (RBC), (**E**) alkaline phosphatase (ALP) and (**F**) aspartate aminotransferase (AST)

Considering the safety of SNO-CS@MoS_2_ for clinical application, a comprehensive in vivo analysis of the biosafety was performed. Histological analyses of major tissues, including the heart, liver, spleen, lung, and kidney, are illustrated in Fig. 9A, showing no damage or abnormal defects after SNO-CS@MoS_2_ + NIR treatment. Besides, the values of the three representative haematological indices concerned (RBC, HGB and WBC) were all within the normal range (Fig. 9B-D). Indicators related to liver function (ALP and AST) were also within the normal range (Fig. 9E-F). Thus, the newly designed SNO-CS@MoS_2_ demonstrated excellent biocompatibility and biosafety, making them suitable for clinical applications. Taken together, these findings suggest that SNO-CS@MoS_2_ has excellent ability to promote healing of infected wounds, which can be attributed to the following factors: (1) antibacterial activity and inflammation control; (2) enhancement of local vascular regeneration; (3) promotion of wound fibrous deposition and epithelialization; (4) excellent biocompatibility. This innovative system holds immense potential in treating skin infections primarily caused by bacteria.

## Conclusion

In this study, we successfully developed a multimodal synergistic antibacterial system (SNO-CS@MoS_2_), intended for the combined treatment of soft tissue infections. The system utilizes easily surface-modified thin-layer MoS_2_ nanosheets as photothermal agents, which are loaded with SNO-CS through electrostatic interactions, thus realizing the combination of NO gas therapy and PTT. This surface modification not only enables SNO-CS@MoS_2_ to serve as a versatile NO gas donor but also improves its long-term photothermal stability and biocompatibility in comparison to pure MoS_2_. The positive surface modification of SNO-CS@MoS_2_ facilitates its adsorption onto bacteria. Subsequently, the application of PTT rapidly triggers the generation of a substantial quantity of NO by SNO-CS@MoS_2_. The abundant NO, combined with the photothermal antibacterial effect, achieves an efficient synergistic antibacterial therapy, by inducing the disruption of the bacterial membrane, leading to bacterial protein leakage, as well as impairing ATP synthesis functions. Notably, the utility of SNO-CS@MoS_2_ does not cease with antibacterial effects. Upon the eradication of bacteria, residual SNO-CS@MoS_2_ continues to release trace amounts of NO in physiological environment. This sustained NO release stimulates fibroblast migration, proliferation, and vascular regeneration, resulting in accelerated wound healing. This study concluded that SNO-CS@MoS_2_, a novel multifunctional system with safe and outstanding antibacterial characteristics and potential for tissue regeneration, has promising applications in infected soft tissue wound treatment.

### Electronic supplementary material

Below is the link to the electronic supplementary material.

Supplementary Material 1

## Data Availability

The datasets used and analyses during the current study are available from the corresponding author on reasonable request.
