# Supplementary material for: Functionalized MoS2-nanosheets with NIR-Triggered nitric oxide delivery and photothermal activities for synergistic antibacterial and regeneration-promoting therapy
Source: J Nanobiotechnology. 2023 Dec 4;21:463. doi: 10.1186/s12951-023-02167-9 (PMC10694958; doi:10.1186/s12951-023-02167-9)
Supplement: Supplementary file 1 — Supplementary Material 1 [file 12951_2023_2167_MOESM1_ESM.docx]

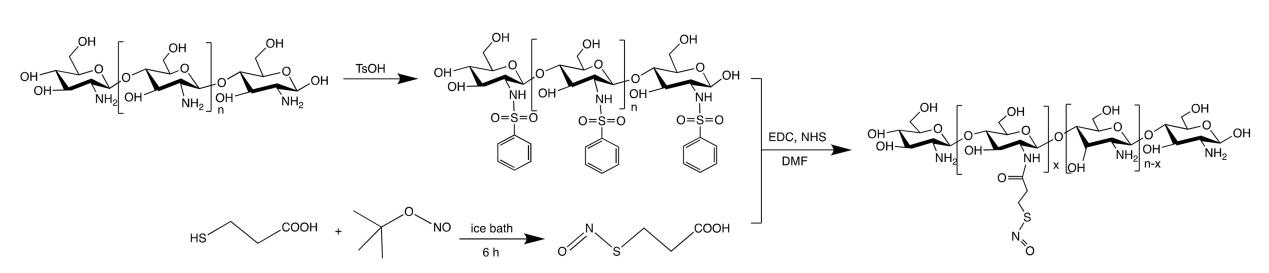


**Fig. S1** A) Synthesis chemical equation of 3-(nitroso) propionic acid and SNO-modified chitosan (SNO-CS).


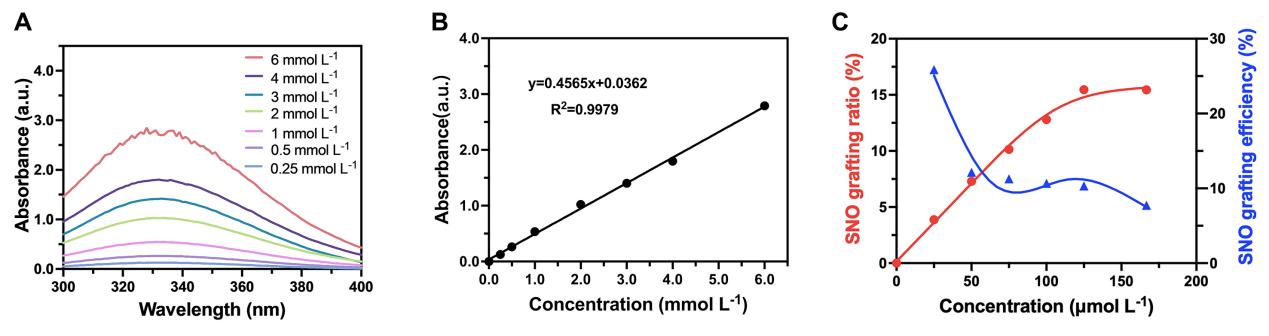


**Fig. S2** A) UV-vis absorbance spectra of 3-(nitroso) propionic acid in different concentration. B) standard curve of 3-(nitroso) propionic acid. C) The grafting ratio and efficiency of 3-(nitroso) propionic acid (SNO) on CS with different concentration of 3-(nitroso) propionic acid.


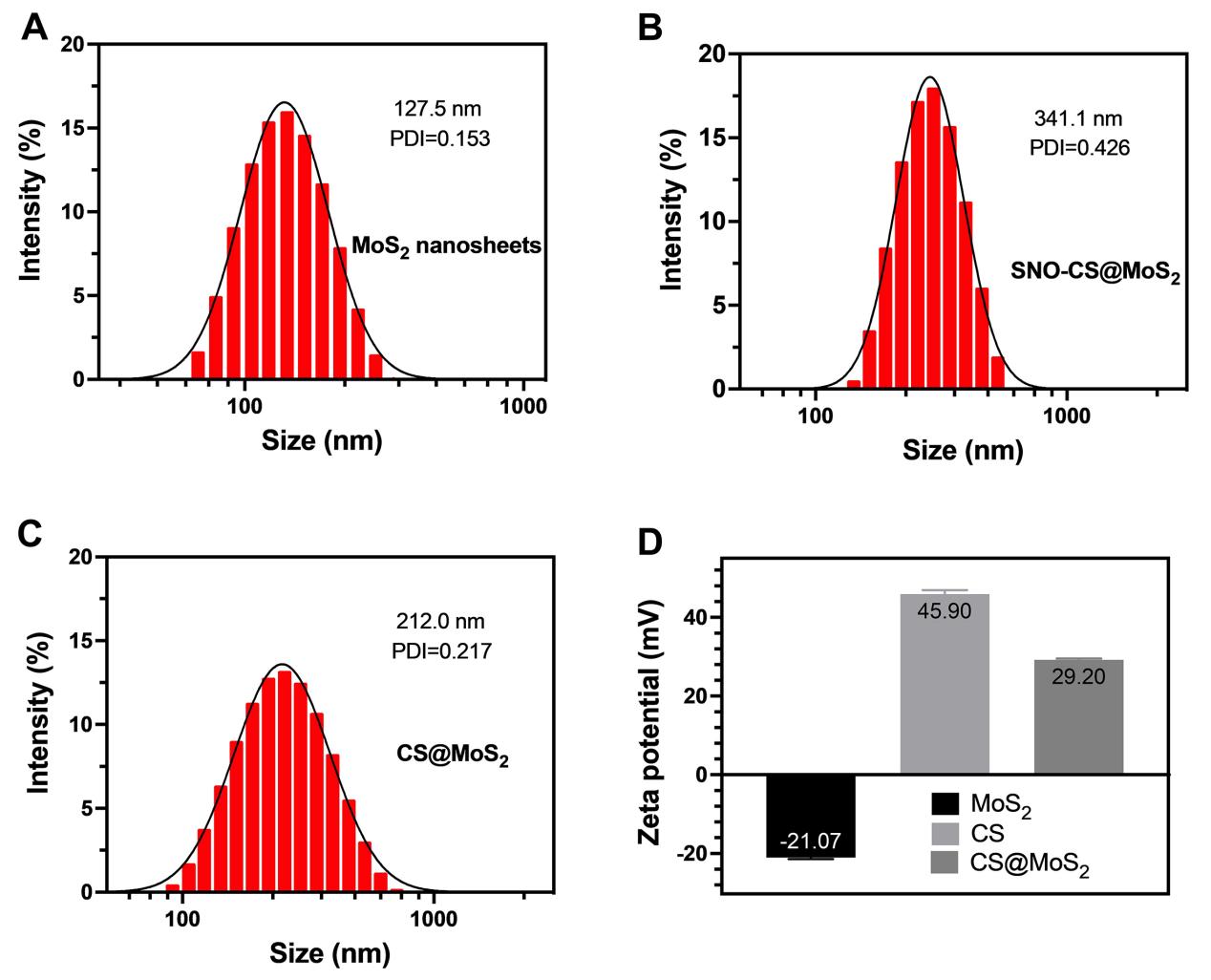


**Fig. S3** Particle size distribution of A) MoS_2_ nanosheets, B) SNO-CS@MoS_2_ nanosheets and C) CS@MoS_2_ nanosheets. D) Zeta potential of CS, MoS_2_ nanosheets and CS@MoS_2_ nanosheets.


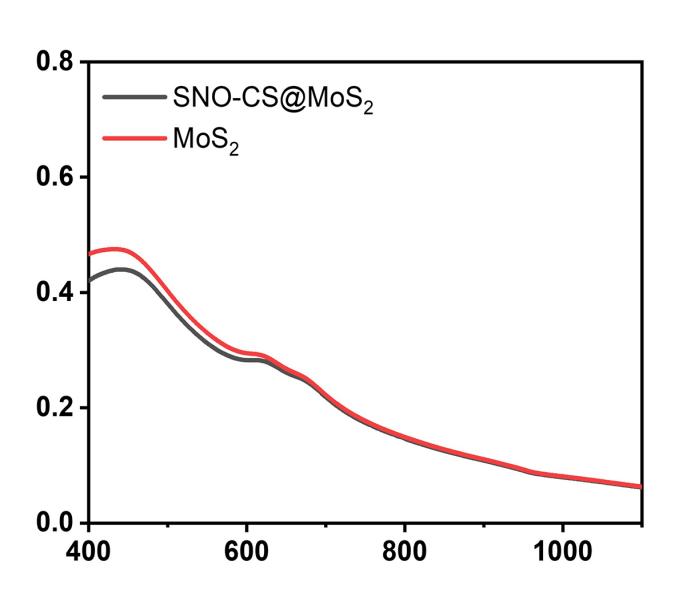


**Fig. S4** UV-vis-NIR spectra of MoS2 and SNO-CS@MoS2


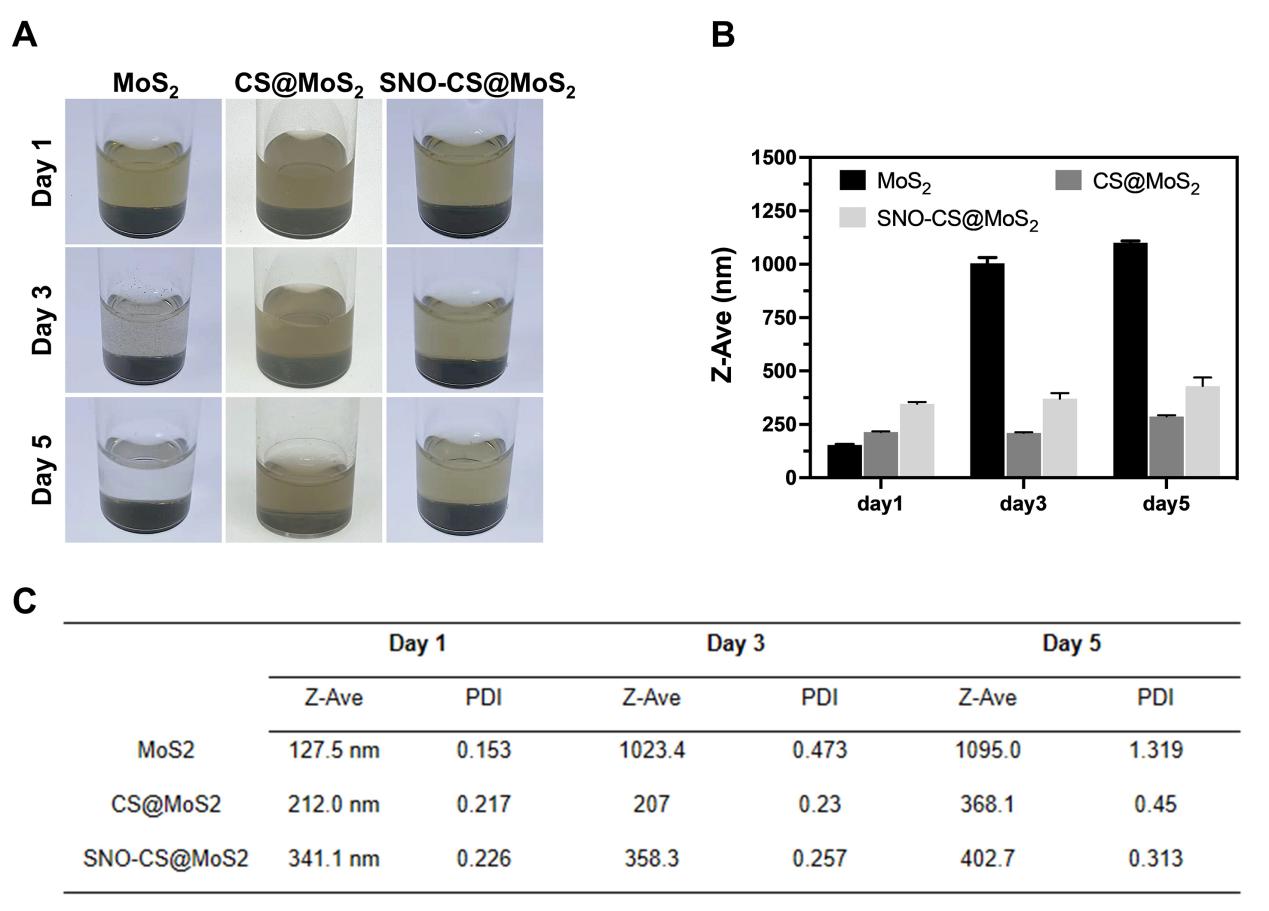


**Fig. S5** A) Photographs and B-C) corresponding particle size changes of MoS_2_, CS@MoS_2_ and SNO-CS@MoS_2_ nanosheets in PBS solution at day 1, 3, 5.


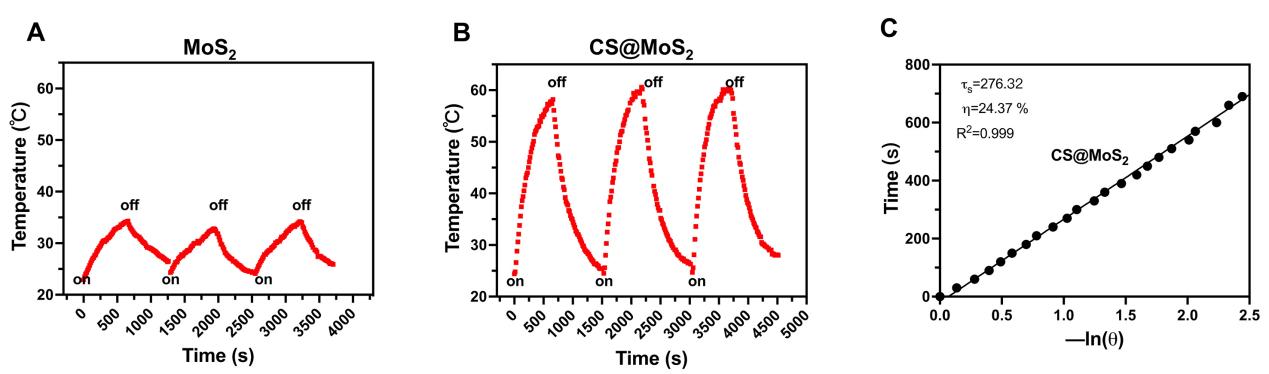


**Fig. S6** Temperature elevations of A) MoS_2_ nanosheets and B) CS@MoS_2_ nanosheets for three NIR light irradiations cycles under an 808 nm laser (200 μg mL^−1^, 1 W cm^−2^). C) The cooling time plot versus −ln(𝜃) of CS@MoS_2_ nanosheets (200 μg mL^−1^).


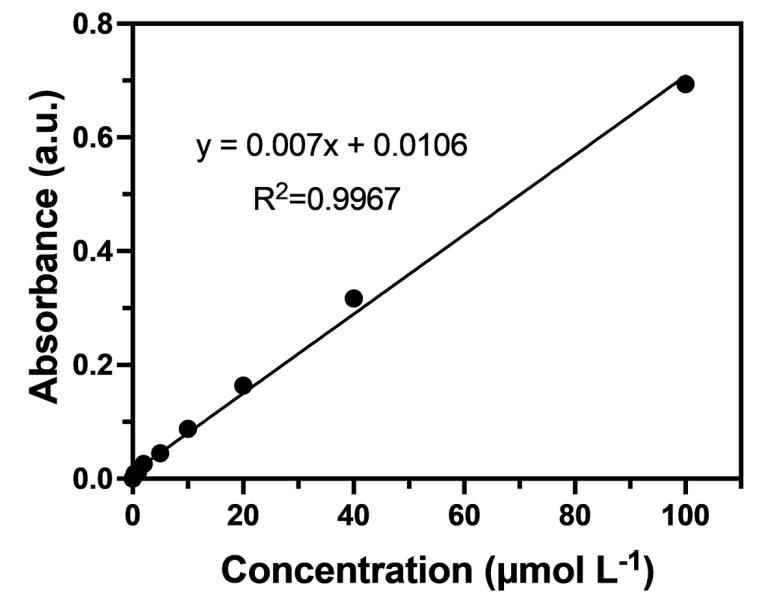


**Fig. S7** Standard curve of NO quantified with the Gress reagent kit.

**
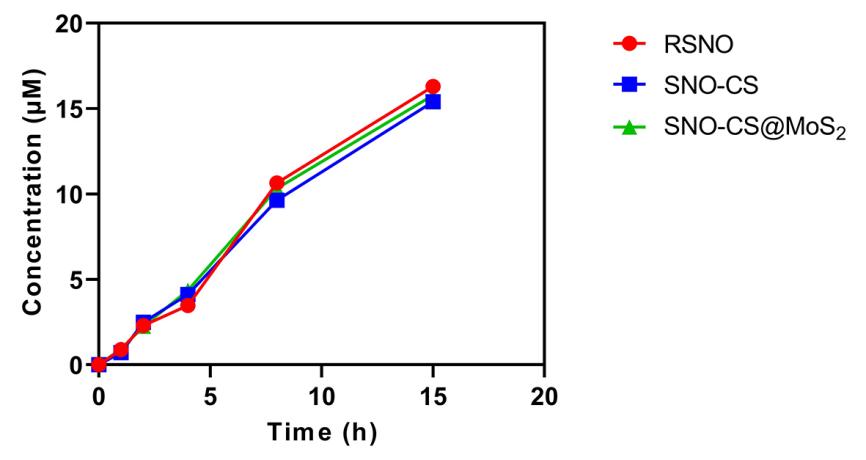
**

**Fig. S8** NO release curves of RSNO, SNO-CS, SNO-CS@MoS_2_ nanosheets in PBS using 37 ℃ natural light.

**
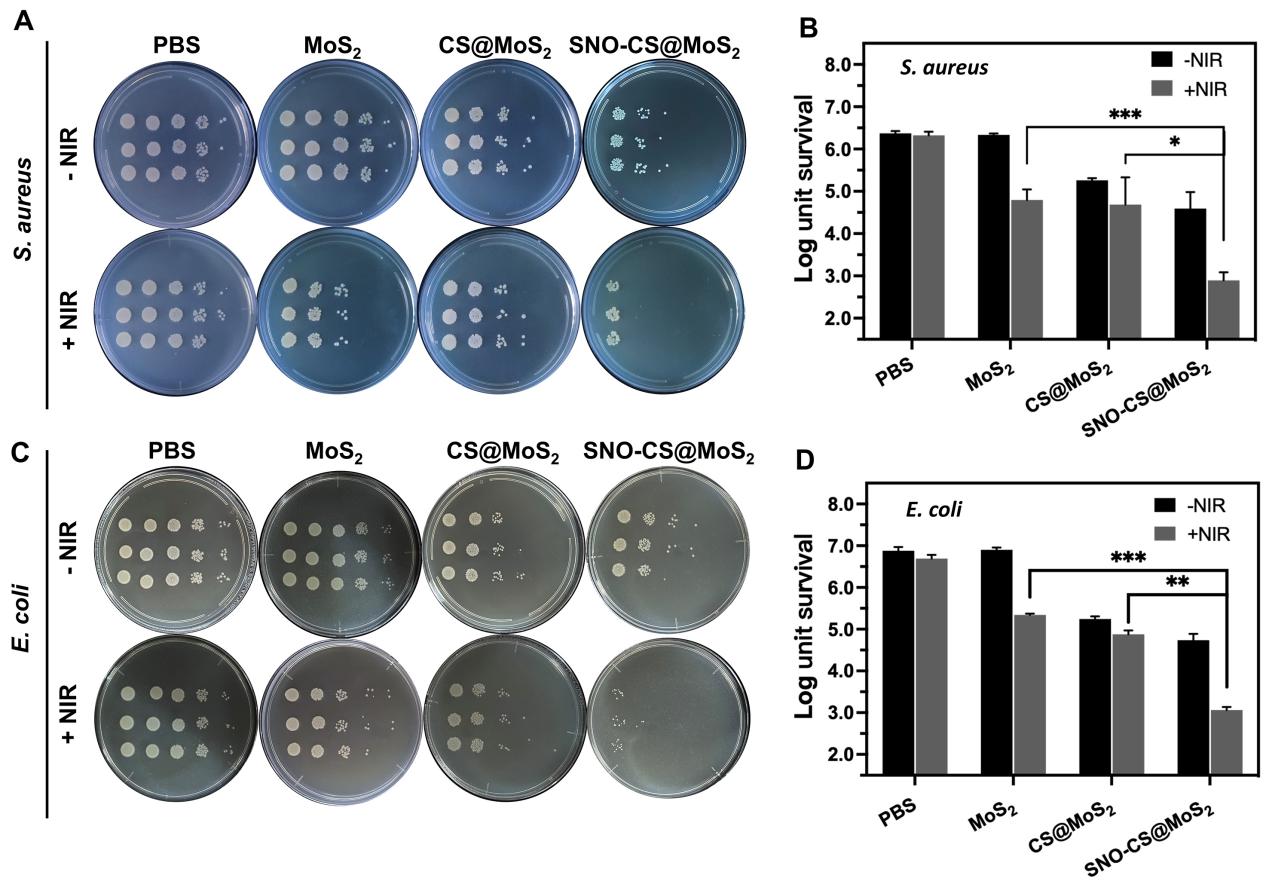
**

**Fig. S9** Photographs of bacterial colonies formed by A) *S. aureus* and C) *E. coli after various treatment*. The corresponding bacterial viabilities of B) *S. aureus*, D) E. coli. (Concentration: 100 μg mL^−1^).


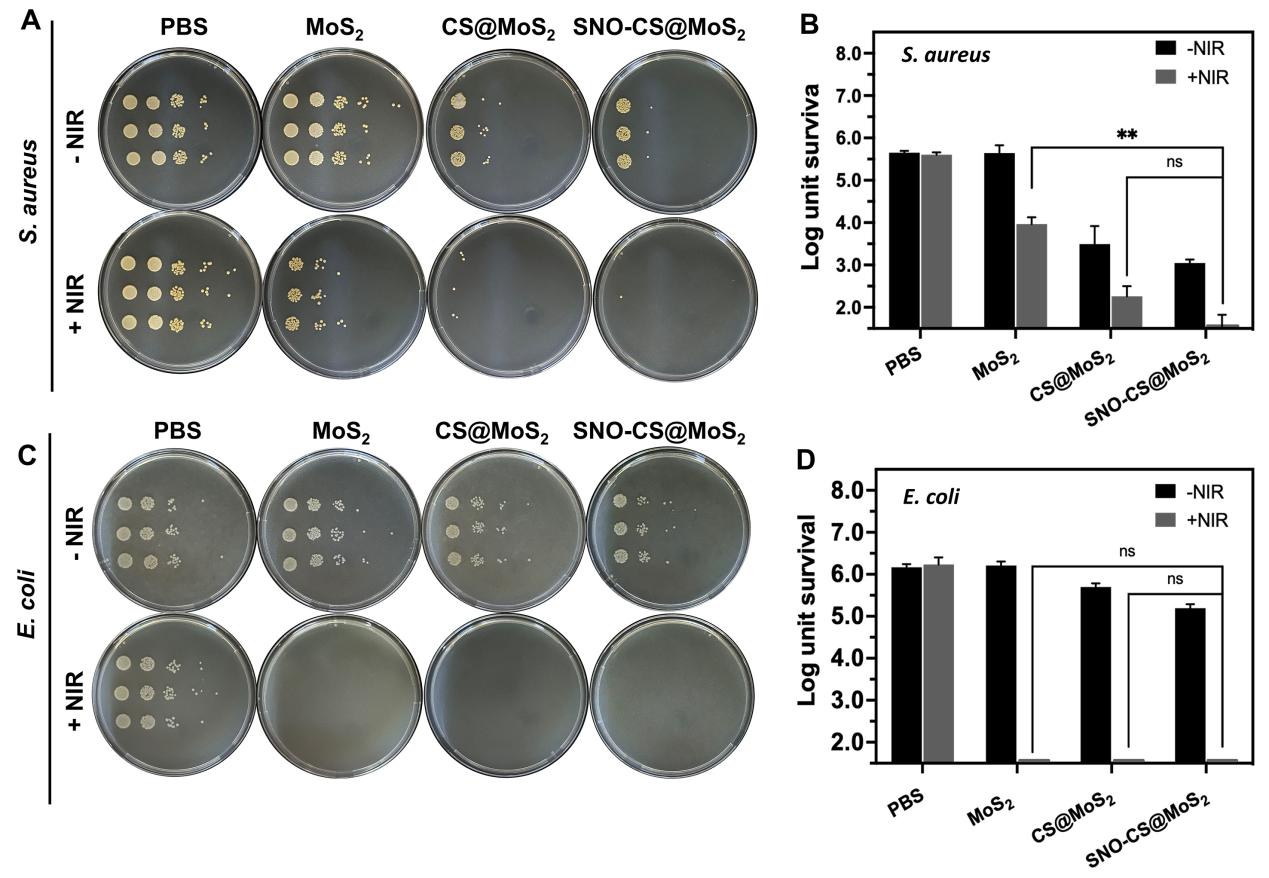


**Fig. S10** Photographs of bacterial colonies formed by A) *S. aureus* and C) *E. coli* after various treatment. The corresponding bacterial viabilities of B) *S. aureus*, D) E. coli. (Concentration: 400 μg mL^−1^).


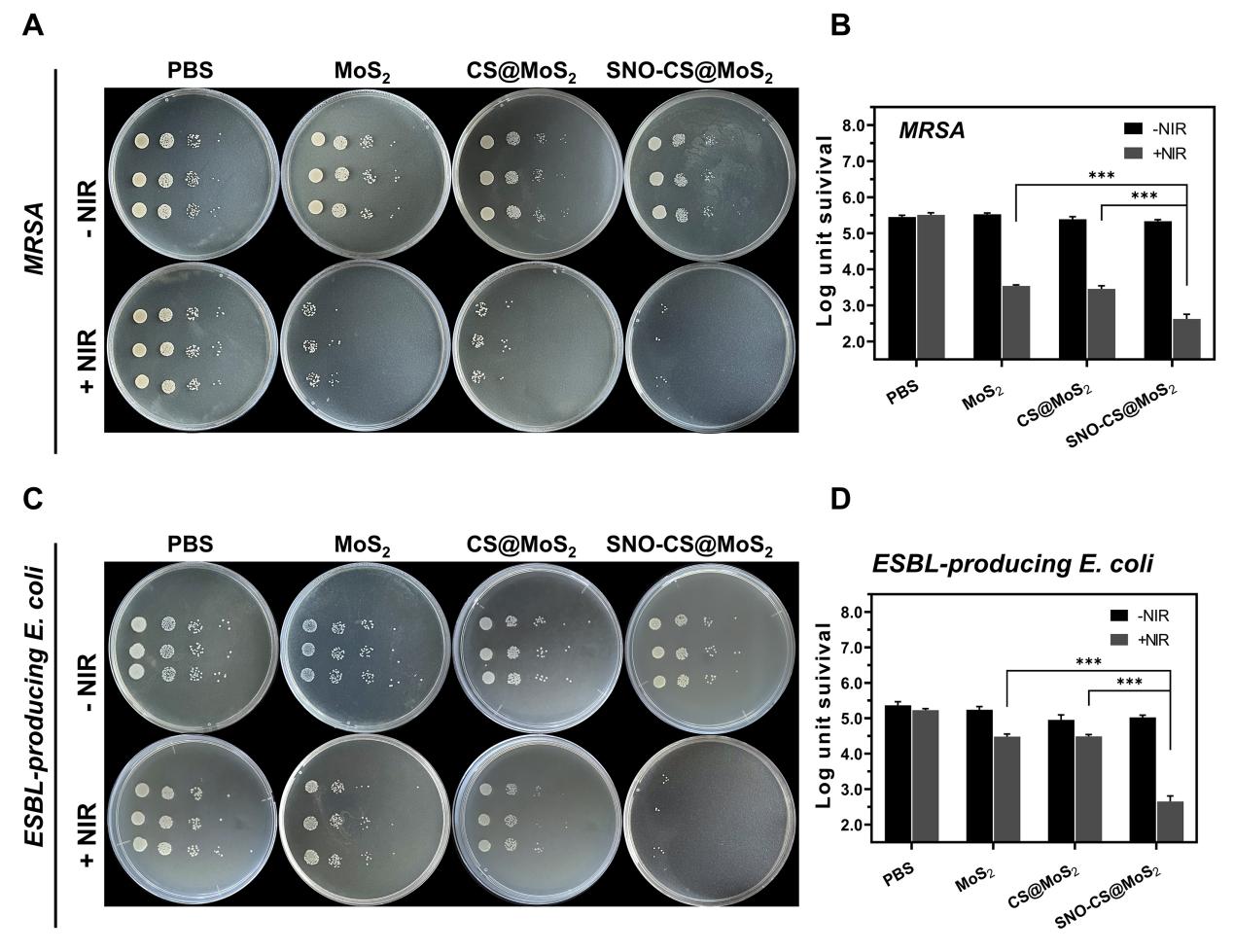


**Fig. S11** Photographs of bacterial colonies formed by A) *MRSA* and C) *ESBL-producing E. coli* after various treatment. The corresponding bacterial viabilities of B) *MRSA*, D) *ESBL-producing E. coli*. (Concentration: 200 μg mL^−1^).


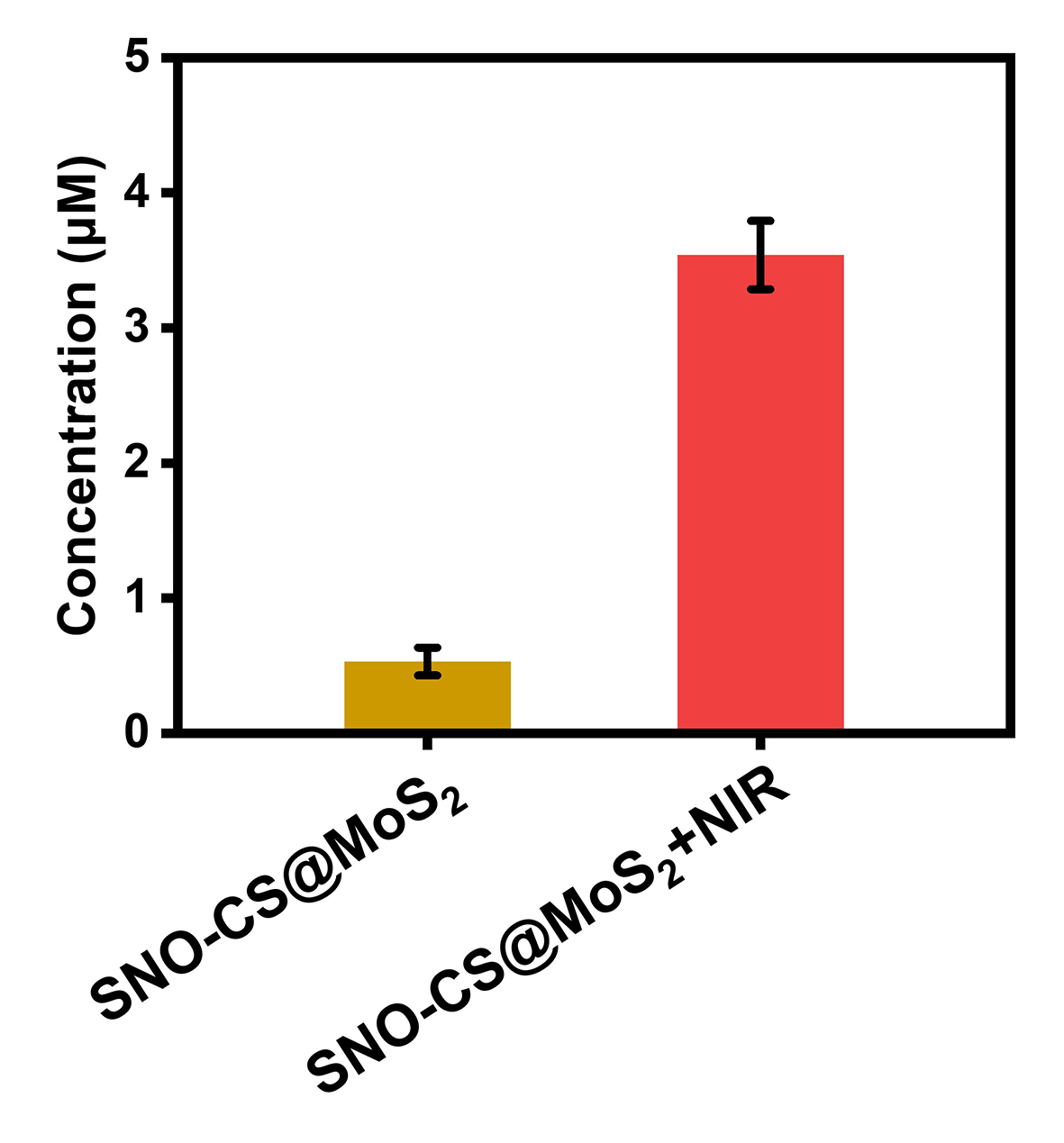


**Fig. S12** NO release curves of SNO-CS@MoS_2_ nanosheets with or without NIR irradiation (808nm, 1 W cm^-2^) *in vivo*.


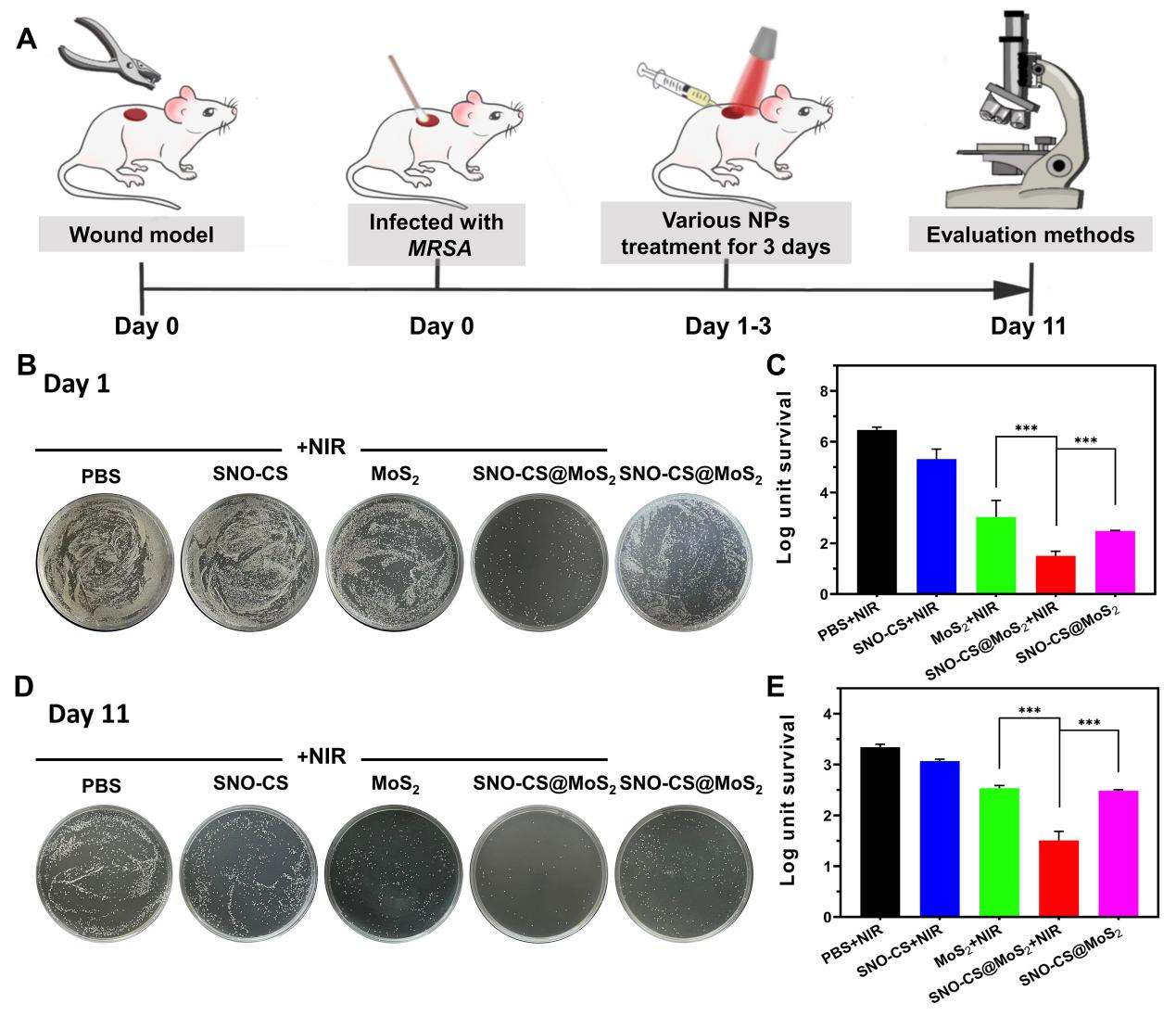


**Fig. S13** A) Schematic illustration for the establishment of an MRSA infected wound model and the subsequent treatment regime. B-C) Cultured bacteria colonies on plates separated from wound tissues after treatment of day 1 and relative survival log of bacteria. D-E) Cultured bacteria colonies on plates separated from wound tissues after treatment of day 11 and relative survival log of bacteria.

**Table S1.** Primer sequence of angiogenesis-related genes

| Gene | Forward primer sequence (5’-3’) | Reverse primer sequence (3’-5’) |
| --- | --- | --- |
| *CD-31* | AACGGAAGGCTCCCTTGATG | TAAGAACCGGCAGCTTAGCC |
| *VEGFα* | ACCACACCATCACCATCGAC | TTCCGGGCTCGGTGATTTAG |

**Table S2.** The weight percentage of each element in the SNO-CS@MoS_2_ system

| Element | C | N | O | S | Mo |
| --- | --- | --- | --- | --- | --- |
| Mass Fraction (%) | 20.43% | 1.14% | 7.68% | 33.21% | 37.54% |
